# Antioxidant Effects and Potential Molecular Mechanism of Action of *Limonium aureum* Extract Based on Systematic Network Pharmacology

**DOI:** 10.3389/fvets.2021.775490

**Published:** 2022-01-05

**Authors:** Zhen Yang, Yanan Mo, Feng Cheng, Hongjuan Zhang, Ruofeng Shang, Xuehong Wang, Jianping Liang, Yu Liu, Baocheng Hao

**Affiliations:** ^1^Key Laboratory of New Animal Drug Project, Lanzhou, China; ^2^Key Laboratory of Veterinary Pharmaceutical Development, Ministry of Agriculture and Rural Affairs, Lanzhou, China; ^3^Lanzhou Institute of Husbandry and Pharmaceutical Sciences of Chinese Academy of Agriculture Sciences, Lanzhou, China

**Keywords:** oxidative stress, network pharmacology, *Limonium aureum* (L.) Hill., antioxidant mechanism, HIF-1 signaling pathway, ErbB signaling pathway

## Abstract

Oxidative stress is the redox imbalance state of organisms that involves in a variety of biological processes of diseases. *Limonium aureum* (L.) Hill. is an excellent wild plant resource in northern China, which has potential application value for treating oxidative stress. However, there are few studies that focused on the antioxidant effect and related mechanism of *L. aureum*. Thus, the present study combining systematic network pharmacology and molecular biology aimed to investigate the antioxidant effects of *L. aureum* and explore its underlying anti-oxidation mechanisms. First, the antioxidant activity of *L. aureum* extracts was confirmed by *in vitro* and intracellular antioxidant assays. Then, a total of 11 bioactive compounds, 102 predicted targets, and 70 antioxidant-related targets were obtained from open source databases. For elucidating the molecular mechanisms of *L. aureum*, the PPI network and integrated visualization network based on bioinformatics assays were constructed to preliminarily understand the active compounds and related targets. The subsequent enrichment analysis results showed that *L. aureum* mainly affect the biological processes involving oxidation-reduction process, response to drug, etc., and the interference with these biological processes might be due to the simultaneous influence on multiple signaling pathways, including the HIF-1 and ERBB signaling pathways. Moreover, the mRNA levels of predicted hub genes were measured by qRT-PCR to verify the regulatory effect of *L. aureum* on them. Collectively, this finding lays a foundation for further elucidating the anti-oxidative damage mechanism of *L. aureum* and promotes the development of therapeutic drugs for oxidative stress.

## Introduction

Oxidative stress is the redox imbalance state of organisms, which is caused by the produced amount of reactive nitrogen or reactive oxygen radicals beyond its scavenging range when organisms are stimulated (1). Many environmental stimuli, including UV, ionizing radiation, chemotherapeutics, heavy metals, and environmental toxins, can trigger the high levels production of reactive oxygen species (ROS) and reactive nitrogen species (RNS), resulting in random oxidative damage of cellular proteins, DNA, and lipids, finally leading to cell death (2, 3). Repeated exposure to oxidative stress accelerates the development of a variety of diseases that include diabetes (4), cancer (5), cardiovascular diseases (6), autoimmune diseases (7), and neurodegenerative disorders (8). Therefore, the balance between ROS production and antioxidant defense helps maintain the normal physiological processes of organisms. Related mechanisms of the redox balance maintenance are critical to the treatment of oxidative stress diseases and have become the research hotspots in recent years.

Network pharmacology is the theory based on systems biology, which was proposed by Hopkins (9, 10) in 2007. Through the integration of multi-disciplinary technologies and contents such as polypharmacology, bioinformatics, and computer science, it constructs the multi-level network of “disease-gene-target-drug” to effectively reveal the relationship between drugs and diseases and elucidate bioinformatics findings and the drug-target-disease mechanisms (11–13). Network pharmacology integrates information obtained by computational methods including graph theory, statistics approaches, data mining, modeling, information visualization, etc., and information obtained by experimental methods including various high-throughput omics techniques, biological and pharmacological experiments, etc. (14). The basic processes of network pharmacology analysis include analysis of candidate ingredients, database construction of target disease, prediction of key targets, network analysis, enrichment analysis, and verification of predicted targets (15–17). As an emerging field based on systems pharmacology, systematic network pharmacology plays an important role in understanding the molecular mechanisms of traditional Chinese medicine in the treatment of complex diseases and is a promising tool for natural drug development.

*Limonium aureum* (L.) Hill. (hereafter referred to as *L. aureum*) is a perennial herb of the Plumbago family. It is a salt-tolerant xerophyte, widely distributed in the Gansu, Xinjiang, Inner Mongolia, Ningxia, and Shanxi regions of China. *Limonium aureum* has the effect of analgesic, anti-inflammatory, blood tonic, detoxification, and anti-oxidation. Decoction of *L. aureum* is used for wind heat cold, neuralgia, less menstruation, tinnitus, lack of milk, headache, toothache, etc. (18–20). As an excellent wild plant resource in northern China, *L. aureum* is easy to develop and utilize without a lot of investment. In view of its analgesic, hemostatic, anti-inflammatory, and antioxidant effects (21–23), it indicates that the development of the active components of *L. aureum* has great potential in drug application. At present, the research on *L. aureum* is mainly focused on the extraction, separation, and structure identification of its effective components (21, 24) and the plant breeding (25). The active substances of *L. aureum* that have been identified were including homoeriodictyol, eriodictyol, naringenin, kaempferol, quercetin, myricetin, luteolin, myricetin-3-O-β-D-glucopyranoside, myricetin-3-O-β-D-galactopyranoside, sitosterol acetate, etc. (21, 24, 26). Among them, quercetin (27), kaempferol (28), myricetin (29), etc. have been proven to have antioxidant effects. However, there were few studies that have been done to reveal the antioxidant mechanism of *L. aureum* from the cellular and molecular level.

**Figure 1:**
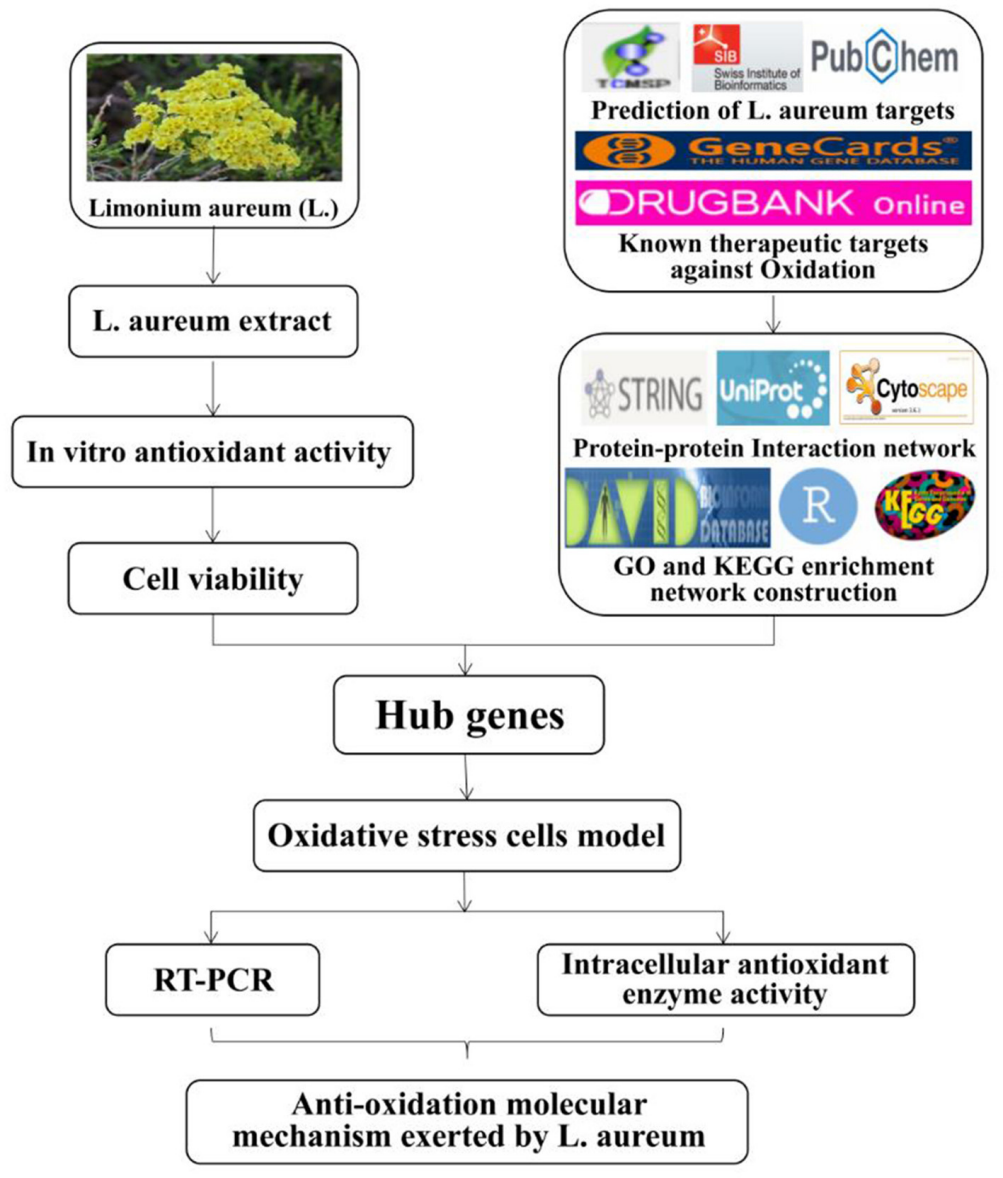
The roadmap of *L. aureum* extract for the treatment of oxidation.

Thus, the aim of the present study was to investigate the antioxidant effects of *L. aureum* and explore its underlying anti-oxidation mechanisms through the combination of systematic network pharmacology and molecular biology. Thus, a “compound-gene-disease” network was constructed through systematic network pharmacology, revealing the regulation mechanism of the active ingredients of *L. aureum* on oxidative stress in a high-throughput manner. Also, the results of network pharmacology were verified by using cell biology methods and techniques including the establishment of cell oxidative stress models, qRT-PCR, etc. To be specific, we first clarified the antioxidant activity of *L. aureum* by using *in vitro* antioxidant assays. Then we detected the effects of *L. aureum* on the RAW264.7 cells viability by CCK-8 method, and evaluate the protective effect of *L. aureum* against oxidative stress in RAW264.7 cells by investigating the intracellular contents of SOD, MDA, LDH, and CAT. Next, we used the online-accessible databases of TCMSP, DrugBank, SwissTargetPrediction, GeneCards, etc. to screen the hub genes of antioxidant and *L. aureum*. The use of STRING database and Cytoscape 3.7.1 software was aiming to perform the protein–protein interaction (PPI) and topological analyses. Furthermore, we performed GO functional and KEGG analyses to identify the mechanism of *L. aureum* in the effect of anti-oxidation. Finally, the mRNA levels of hub genes were measured by quantitative real-time PCR to verify the regulatory effect of *L. aureum* on hub genes. The roadmap was demonstrated by using bioinformatics and computational analyses to reveal target-based and pathway-based prioritization in *L. aureum* treated oxidation (Figure 1). The systematic network pharmacology method was combined with cell biological approaches in this research, hoping to deepen the understanding of the effective, potential active ingredients of *L. aureum* for anti-oxidation and reveal the pharmaceutically acceptable targets, thereby promoting the development of effective anti-oxidative therapeutic drugs.

## Materials and Methods

### Materials and Reagents

The whole plants of the *Limonium aureum* (L.) Hill. species were harvested in August 2020 at Dawa Mountain Comprehensive Experimental Base of Lanzhou Institute of Husbandry and Pharmaceutical Sciences of CAAS, Gansu Province, China. The sample was identified by Fuping Tian (Prataculture research group of Lanzhou Institute of Husbandry and Pharmaceutical Sciences of CAAS, Gansu Province, China). Ethylene diamine tetraacetic acid disodium salt (EDTA-2Na) and ascorbic acid (Vc) were purchased from Sinopharm Chemical Reagent Co. (Shanghai, China), and 2,2-diphenyl-1-picrylhydrazyl (DPPH) was purchased from TCI (Shanghai) Development Co. Ltd. Other chemical reagents used in experiments including hydrogen peroxide (H_2_O_2_), ferrous sulfate (FeSO_4_), salicylic acid, ethanol, iron (II) chloride tetrahydrate (FeCl_2_·4H_2_O), ferrozine, etc. were bought from local suppliers. All the chemical reagents used were analytical grade. Cell Counting Kit-8 (CCK-8) was obtained from Biosharp Life Sciences (Hefei, China). Fetal bovine serum and Dulbecco's modified Eagle's medium (DMEM) high glucose were purchased from Gibco Life Technology (New York, USA) and HyClone (Utah, USA). SOD, CAT, LDH, and MDA assay kits were obtained from Solarbio Science & Technology Co. Ltd. (Beijing, China). Simply P total RNA extraction kit was purchased from Bioer Technology Co. Ltd. (Hangzhou, China). PrimeScript RT reagent kit with gDNA eraser and TB Green Premix Ex Taq II were purchased from Takara Bio (Japan). Murine macrophage cell line RAW264.7 cells were provided by Cell Culture Center of the Chinese Academy of Sciences (Shanghai, China).

### Preparation of *L. aureum* Extract

*L. aureum* was collected from Dawa Mountain Comprehensive Experimental Base of Lanzhou Institute of Husbandry and Pharmaceutical Sciences of CAAS. The collected fresh plants were air-dried avoiding light at room temperature for a week. The dried plants were ground to powder for later extract. *Limonium aureum* (50 g) powder was soaked in 95% ethanol for 2 h and then accelerated the dissolution with ultrasound for 1 h; this procedure was repeated six times. The combined extracts were concentrated in a vacuum rotary evaporator.

### *In vitro* Antioxidant Activity Assay

#### DPPH Radical Scavenging Assay

The DPPH radical scavenging activity of *L. aureum* was assayed according to a previous procedure with minor modifications (30). Briefly, 200 μl of 1 mM DPPH solution (dissolved in 75% ethanol) was prepared and was mixed with 100 μl of various concentrations of *L. aureum* or ascorbic acid (Vc) in 96-well plates. The mixture was placed in the dark at room temperature for 30 min, and the corresponding absorbance (A_1_, A_2_) at 517 nm was recorded by using a spectrophotometer (Epoch Microplate Spectrophotometer; BioTek Instruments, Inc., USA). In addition, A_0_ was the absorbance of the control group (solvent instead of the sample solution). A_1_ was the absorbance of the test group. A_2_ was the absorbance of the sample (75% ethanol instead of the DPPH solution). Ascorbic acid (Vc) was used as the positive control. The DPPH radical scavenging activity of *L. aureum* was calculated by the following formula:


$$\begin{array}{l} \text{DPPH radical scavenging activity }\left(\%\right)\\ =\left[1-\left({\text{A}}_{1}-{\text{A}}_{2}\right)/{\text{A}}_{0}\right]\times 100\% \end{array}$$

#### Hydroxyl Radical Scavenging Assay

The hydroxyl radical scavenging activity of *L. aureum* was evaluated using a previously described procedure (31) with some modifications. Fifty microliters of different concentrations of *L. aureum* and ascorbic acid (Vc) were removed in 96-well plates, respectively. Volumes of 50 μl of 9 mM FeSO_4_ aqueous solution, 50 μl of 9 mM salicylic acid–ethanol solution, and 50 μl of 3.8 mM H_2_O_2_ were added successively and mixed evenly. The mixture was incubated at 37°C for 30 min, and the corresponding absorbance (A_1_, A_2_) at 510 nm was recorded by using a spectrophotometer (Epoch Microplate Spectrophotometer; BioTek Instruments). In addition, A_0_ was the absorbance of the control group (solvent instead of the sample solution). A_1_ was the absorbance of the test group. A_2_ was the absorbance of the sample (distilled water instead of the *L. aureum* solution). Ascorbic acid (Vc) was used as the positive control. The hydroxyl radical scavenging activity of *L. aureum* was calculated by the following formula:


$$\begin{array}{l} \text{Hydroxyl radical scavenging activity }\left(\%\right)\\ =\left[1-\left({\text{A}}_{1}-{\text{A}}_{2}\right)/{\text{A}}_{0}\right]\times \;100\% \end{array}$$

#### Ferrous Ion-Chelating Ability

The ferrous ion-chelating ability was determined according to an earlier reported method (32) with slight modifications. A volume of 100 μl of various concentrations of *L. aureum* and EDTA-2Na were mixed with 5 μl of 4 mM FeCl_2_·H_2_O and 20 μl of 5 mM ferrozine in 96-well plates. A volume of 75 μl distilled water was added to each well after standing at room temperature for 10 min. The absorbance was measured at 560 nm with a spectrophotometer (Epoch Microplate Spectrophotometer; BioTek Instruments). Here, A_0_ was the absorbance of the control group (solvent instead of the sample solution). A_1_ was the absorbance of the test group. A_2_ was the absorbance of the sample (distilled water instead of ferrozine solution). EDTA-2Na was chosen as the positive control to evaluate iron ion-chelating activity. The ferrous ion-chelating ability of *L. aureum* was calculated by the following formula:


$$\begin{array}{l} \text{Ferrous ion-chelating ability }\left(\%\right)\\ =\left[1-\left({\text{A}}_{1}-{\text{A}}_{2}\right)/{\text{A}}_{0}\right]\times \;100\% \end{array}$$

#### Ferric Reducing Power

The ferric reducing power was determined following the method described by Katanic et al. (33) with modifications. Specifically, 100 μl of different dilutions of *L. aureum* and ascorbic acid (Vc) were added to 250 μl phosphate buffer (pH 6.6) and 250 μl potassium ferricyanide (1 wt %). The mixture was incubated in a water bath at 50°C for 20 min. A volume of 250 μl trichloroacetic acid solution (10 wt %) was added to the mixture and centrifuged at 4,000 rpm for 10 min. Supernatant (50 μl) was taken from 96-well plates, and mixed with 50 μl distilled water and FeCl_3_ (1 wt %). The absorbance was measured at a wavelength of 700 nm with a spectrophotometer (Epoch Microplate Spectrophotometer; BioTek Instruments). Here, A_1_ was the absorbance of the test group. A_2_ was the absorbance of all the reagents where distilled water was used instead of FeCl_3_ solution. The ferric reducing power of *L. aureum* was calculated by the following formula:


$$\begin{array}{l} \text{Ferric reducing power}={\text{A}}_{1}-{\text{A}}_{2} \end{array}$$

### Cell Culture

The RAW264.7 cells were provided by Cell Culture Center of the Chinese Academy of Sciences (Shanghai, China). Cells were cultured in DMEM supplemented with 10% fetal calf serum (HyClone, USA) at 37°C in a fully humidified incubator containing 5% CO_2_.

### Cell Viability Assay

The cell viability was measured using the CCK-8 assay according to the manufacturer's instructions and the hydrogen peroxide induced RAW264.7 cells oxidative stress model was established following the method described by Zhou et al. with some modifications (34). RAW264.7 cells were seeded in 96-well plates (Eppendorf, Germany) at a density of 6 × 10^4^/ml in culture medium for 4 h. Subsequently, the cells were incubated with DMEM containing different concentrations of *L. aureum* extracts (dissolved in DMSO) or H_2_O_2_ for 24 h. The viability of cells stimulated with *L. aureum* under oxidative stress were determined as follows: cells were plated in 96-well plates (Eppendorf, Germany) with a density of 1 × 10^5^ in culture medium for 4 h, and the cells were incubated with DMEM containing different concentrations of *L. aureum* extracts (0, 2, 5, 10 μg/ml) for 20 h. The positive control group and the *L. aureum*–treated groups were then exposed to H_2_O_2_ (400 μM) for 4 h. CCK-8 solution (10 μl) was added to each well and incubated in an atmosphere of 5% CO_2_ at 37°C for 4 h. The absorption values were measured at 450 nm by using a spectrophotometer (Epoch Microplate Spectrophotometer; BioTek Instruments). The results were expressed as the percentage viability according to the following formula:


$$\begin{array}{l} \text{Cell viability }(\%)=[(\text{absorbance of treatment}\\ -\text{ absorbance of blank})/(\text{absorbance of control}\\ -\text{ absorbance of blank})]\times 100\% \end{array}$$

### Evaluation of Enzyme Activity and Lipid Peroxidation

RAW264.7 cells were seeded in 6-well plates (Eppendorf, Germany) at a density of 10^6^/ml and cultivated as mentioned previously. The cells were stimulated with *L. aureum* extracts (0, 2, 5, 10 μg/ml) for 20 h. The positive control group and *L. aureum*–treated groups were then exposed to H_2_O_2_ (400 μM) for 4 h. The activity of catalase (CAT), lactate dehydrogenase (LDH), malondialdehyde (MDA), and superoxide dismutase (SOD) in cells was determined using a commercial kit according to the manufacturer's instructions (Beijing Solarbio Science & Technology Co., Ltd., China).

### Network Pharmacology Analysis

#### Screening for Active Ingredients of *L. aureum*

All the chemical constituents of *L. aureum* were obtained by literatures and Traditional Chinese Medicine Systems Pharmacology (35) (TCMSP (36), available online (https://www.tcmsp-e.com/, key word: “BU XUE CAO,” last updated in May 2014). According to oral bioavailability (OB) and drug-likeness (DL), the screening thresholds of each chemical component were set as OB ≥ 30% and DL ≥ 0.18, respectively. The SMILE structures of bioactive ingredients were obtained through PubChem database (available online: https://pubchem.ncbi.nlm.nih.gov/, last updated in March 2019) (37) and their corresponding targets were screened out through SwissTargetPrediction database (available online: http://www.swisstargetprediction.ch/) (38) for subsequent analysis. The target names were converted into gene names by UniProt protein database (available online: https://www.uniprot.org/, last updated in February 2021) (39).

#### Construction of a Bioactive Component-Target Network

The keyword “antioxidant” was used to search disease-related genes on GeneCards database (40) (available online: https://www.genecards.org/, last updated in October 2021) (41), and the antioxidant-related genes were collected with the setting of relevance score of ≥30. The bioactive ingredients targets of *L. aureum* were mapped to the target genes related to antioxidant to obtain the common target genes through the online tool “jvenn” (available online: http://jvenn.toulouse.inra.fr/app/example.html).

#### Protein–Protein Interaction Network Construction

To further elucidate the potential mechanism underlying the antioxidation effect of *L. aureum*, the overlapping antioxidation-related and predicted targets of *L. aureum* were used to construct a protein–protein interaction (PPI) network on STRING database (42, 43) (available online: https://string-db.org/) (44). The protein interaction information including the node degree value was obtained with the set conditions of “Homo sapiens,” “Minimum required interaction score = 0.4,” and “Hide disconnected nodes in the network.” The PPI network was visualized by using Cytoscape 3.7.2 software (45), and based on the obtained node degree values, the “Network Analysis” plug-in was used to analyze the topological properties of each node for the selection of core targets.

#### Gene Ontology and KEGG Pathway Enrichment Analyses

To analyze the biological pathways of genes in the PPI network (43, 46), the computational R-language package of “clusterProfiler (47)” (version 4.1.0) was applied to analyze Gene Ontology (GO) enrichment in biological function/process (BP), cellular component (CC), and molecular function (MF) (adjusted to *p* < 0.05). The DAVID database (available online: https://david.ncifcrf.gov/home.jsp, last updated in May 2016) (48) was used to analyze Kyoto Encyclopedia of Genes and Genomes (KEGG) pathway enrichment with the “Homo sapiens” setting (adjusted to *p* < 0.05). The visualization bubble chart and histogram were formed through “ggplot2” package in R (version 3.3.3).

### Total mRNA Extraction and qRT-PCR

The extraction of total RNA from cells treated with *L. aureum* (10, 5, 2 μg/ml) and H_2_O_2_ (400 μM) was performed using the Simply P Total RNA Extraction Kit (Bioflux, Hangzhou, China). Total RNA was reverse-transcribed into cDNA using PrimeScript RT reagent Kit with gDNA Eraser (Perfect Real Time) (Takara, Japan). The quantitative real-time polymerase chain reaction (qRT-PCR) and QuantStudio (Thermo Fisher, USA) with TB Green Premix Ex Taq II (Takara, Japan) were applied to the PCR-amplified hub genes. β-Actin served as the internal control. The primers for the hub genes are listed in Supplementary Table S1. The relative mRNA expression was calculated according to 2^−ΔΔCT^.

### Statistical Analysis

Experimental data were expressed as the mean ± SD of three independent experiments. The one-way ANOVA in SPSS 26.0 for Windows (SPSS Inc., Chicago, IL) was used for statistical analysis. *P*-values <0.05 were considered statistically significant.

## Results

### *In vitro* Antioxidant Activity

#### Scavenging Activity Against DPPH Free Radical

**Figure 2:**
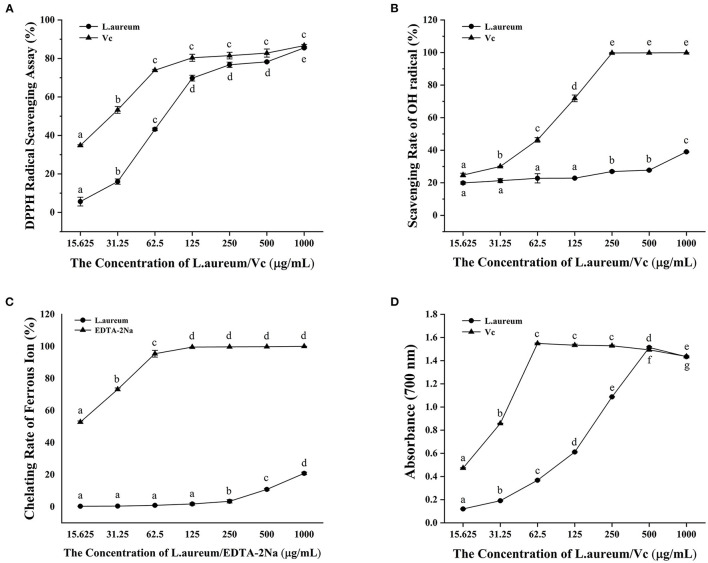
*In vitro* antioxidant activity of *L. aureum*. **(A)** Scavenging activity against the DPPH radical. **(B)** Scavenging activity against the OH radical. **(C)** Ferrous ion-chelating ability. **(D)** Ferric reducing power. The significant difference (*p* < 0.05) in the same sample is indicated by different letters.

DPPH free radical scavenging ability is widely used to evaluate the total free radical scavenging ability of substances. It is a stable free radical, which accepts an electron or hydrogen radical that can be reduced to yellow diphenyl-picrylhydrazine. The DPPH free radical scavenging ability can be determined according to the degree of absorbance reduction, which is affected by the antioxidants (49). As illustrated in Figure 2A, both *L. aureum* and vitamin C had strong scavenging activity against DPPH free radical in a concentration-dependent manner. The DPPH radical scavenging activity of *L. aureum* and vitamin C increased quadratically when the concentration of *L. aureum* and vitamin C increased from 15.625 to 125 μg/ml. Compared with *L. aureum*, vitamin C had stronger scavenging ability against DPPH in doses ranging from 15.625 to 250 μg/ml (*p* < 0.05). At the doses of 500 and 1,000 μg/ml, the DPPH scavenging ability of *L. aureum* and vitamin C had no significant difference (*p* > 0.05).

#### Scavenging Activity Against Hydroxyl Free Radical

The hydroxyl radical is one of the most active free radicals that can react with all biological macromolecules in living cells (50). The capacity of *L. aureum* to scavenge hydroxyl free radical generated by the Fenton reaction between Fe^2+^ and H_2_O_2_ was evaluated (51). As shown in Figure 2B, we found that vitamin C and *L. aureum* can effectively scavenge hydroxyl free radical. The hydroxyl scavenging effect of vitamin C and *L. aureum* increased with the increases of their concentration. When the concentration of vitamin C reached 250 μg/ml, vitamin C can effectively scavenge almost 100% hydroxyl radical. Meanwhile, the scavenging effect of *L. aureum* in all concentrations was lower than 50%.

#### Ferrous Ion-Chelating Ability

The hydroxyl radicals are generated through Fenton reaction, which accelerates the lipid peroxidation chain reaction (52). Chelating agents can inhibit OH formation by forming complexes with ferrous ions, thereby exerting their antioxidant effect (53). Thus, the ferrous ion-chelating ability of *L. aureum* was measured in this study. Results showed that *L. aureum* have low Fe^2+^ ion-chelating capacity (Figure 2C). When the concentration was lower than 250 μg/ml, the chelating ability of *L. aureum* to ferrous ions was almost 0. When the concentration reached 1,000 μg/ml, the chelating ability of *L. aureum* to ferrous ions was 20.85%, while EDTA-2Na, which was the positive control, had an iron chelating capacity of 100% at this concentration. The results indicated that the scavenging effect of *L. aureum* on hydroxyl free radicals might be caused by direct quenching of hydroxyl free radicals, rather than by scavenging free ferrous ions.

#### Ferric Reducing Power

Potassium ferricyanide (Fe^2+^) is formed by the reaction between substance with reduction potential and potassium ferricyanide (Fe^3+^) reacts with ferric chloride to form an iron trivalent complex, which has a maximum absorption at 700 nm (54). Ferric reducing power of *L. aureum* was evaluated by its ability to reduce Fe^3+^ (CN^−^)_6_ to Fe^2+^ (CN^−^)_6_, which was determined by monitoring the absorbance of the complex formed after the addition of ferric chloride. A dose-dependent ferric reducing power of vitamin C and *L. aureum* was observed in Figure 2D. The ferric reducing power of *L. aureum* increased linearly when its concentration is <500 μg/ml. While the concentration exceeded 500 μg/ml, its ferric reducing power decreased significantly (*p* < 0.05). Moreover, the difference in ferric reducing power of vitamin C and *L. aureum* at concentrations of 500 and 1,000 μg/ml was not significant (*p* < 0.05).

### The Effects of *L. aureum* and H_2_O_2_ on the Viability of RAW264.7 Cells

**Figure 3:**
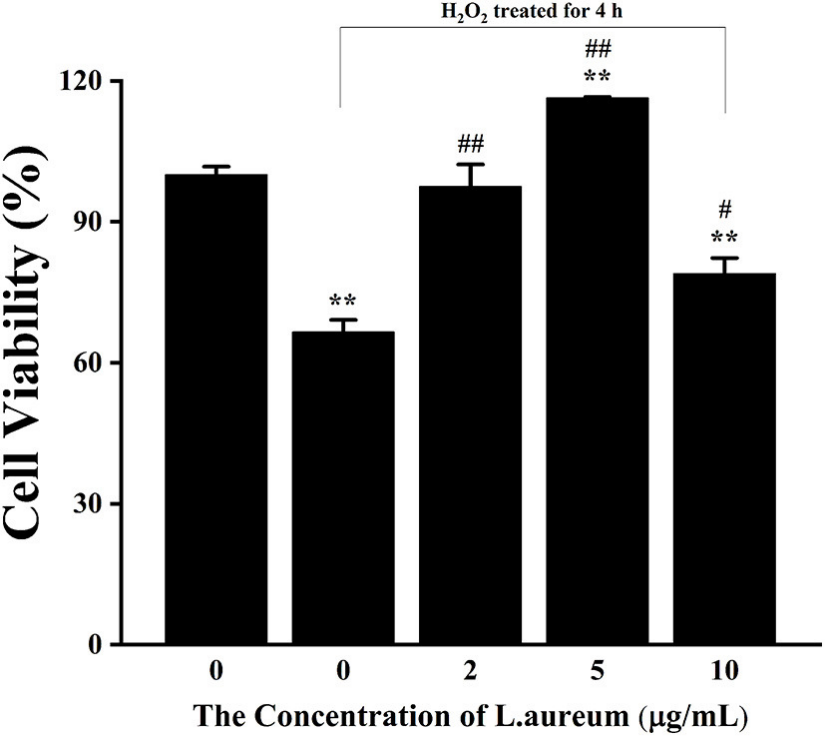
The effect of *L. aureum* on the viability of RAW264.7 cells under oxidative stress. Cells were treated with different concentrations of *L. aureum* (0, 2, 5, 10 μg/ml) for 20 h. The positive control group and *L. aureum* treated groups were then exposed to H_2_O_2_ (400 μM) for 4 h. The results were expressed as the mean ± SD of three independent experiments. **p < 0.01 compared with blank control group, #*p* < 0.05 and ##*p* < 0.01 compared with positive control group were considered statistically significant differences.

The effects of different concentrations of H_2_O_2_ of and *L. aureum* on cell viability were determined by using the CCK-8 analysis. As shown in Supplementary Figure S1A, the viability of RAW264.7 was gradually decreased with the increasing concentration of H_2_O_2_. The viability of cells was significantly inhibited when the concentration of H_2_O_2_ reached 200 μM (*p* < 0.01). According to the determined IC_50_ (340.12 μM), H_2_O_2_ with a concentration of 400 μM was selected for the following experiments. As shown in Supplementary Figure S1B, the viability of cells was significantly decreased when the concentration of *L. aureum* reached 20 μg/ml (*p* < 0.01), which indicated *L. aureum* had cytotoxicity at 20 μg/ml, while it was not toxic to cells in the concentration range of 1–10 μg/ml. Moreover, the significant increases of cell viability were shown when the concentrations of *L. aureum* were 2 μg/ml (*p* < 0.01) and 5 μg/ml (*p* < 0.05), respectively. It was speculated that within this concentration range, *L. aureum* might have a certain promoting effect on cell proliferation. Therefore, 2, 5, and 10 μg/ml were selected to be the low, medium, and high dose of *L. aureum* in the subsequent experiments (Figure 3).

### Changes of Enzyme Activity in H_2_O_2_-Induced RAW264.7 Cells

**Figure 4:**
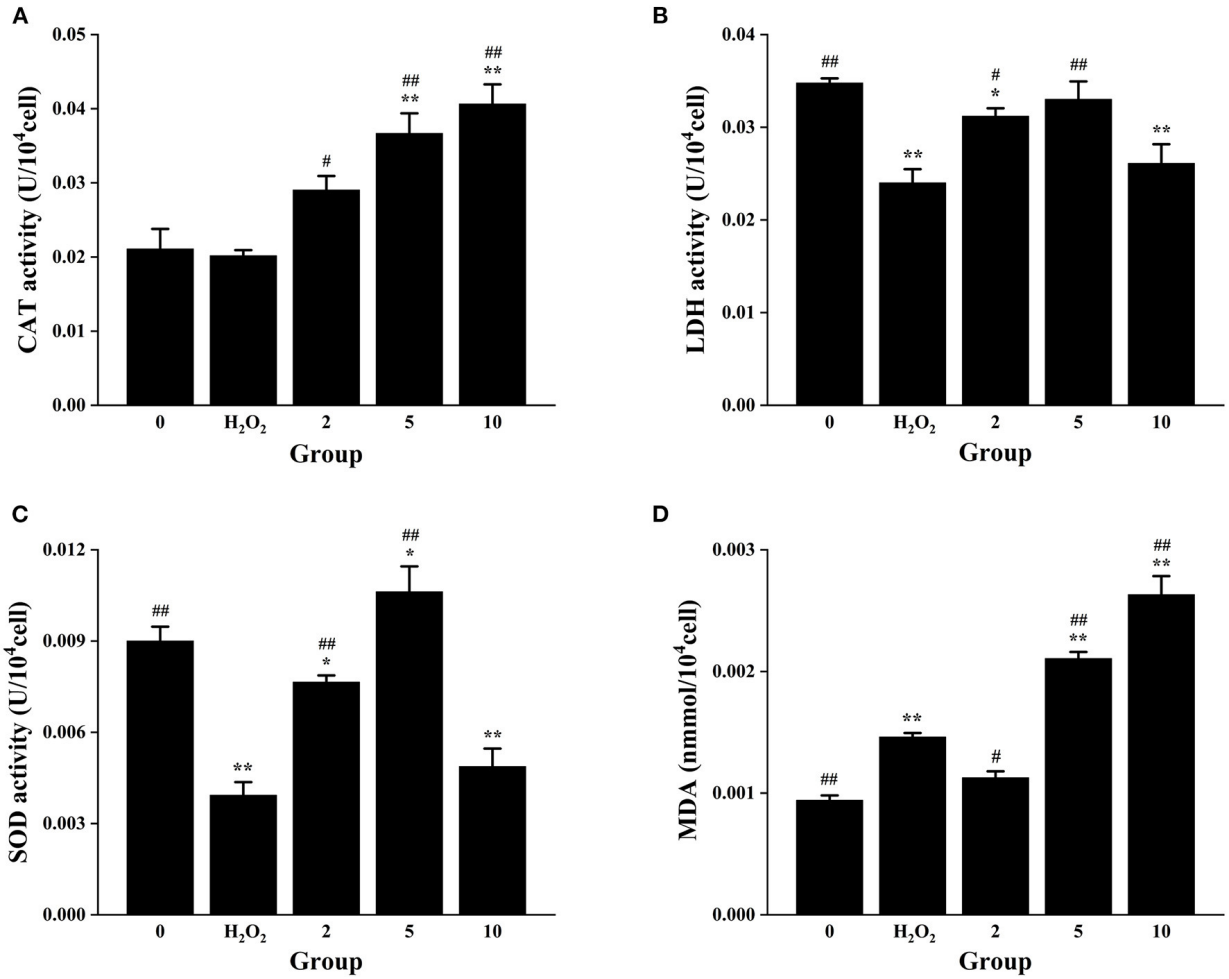
Effects of *L. aureum* on the antioxidant enzyme activity and lipid peroxidation in H_2_O_2−_induced RAW264.7 cells. **(A)** The effect on the activity of CAT. **(B)** The effect on the activity of LDH. **(C)** The effect on the activity of SOD. **(D)** The effect on the cellular MDA level. The results are expressed as the mean ± SD of three independent experiments. *p < 0.05 and **p < 0.01 compared with H_2_O_2_-untreated group, #*p* < 0.05 and ##*p* < 0.01 compared with H_2_O_2_ group were considered statistically significant differences.

For the purpose of further understanding the antioxidant activity of *L. aureum*, the effects of *L. aureum* on the antioxidant enzyme activity and lipid peroxidation in H_2_O_2_-induced RAW264.7 cells were evaluated. As presented in Figure 4A, the CAT activity of middle- and high-dose *L. aureum*–treated groups significantly increased compared with the H_2_O_2_ group (*p* < 0.01). As can been seen in Figures 4B,C, the LDH activity and SOD activity showed similar tendency—the LDH activity and SOD activity were significantly increased in the low- and middle-dose *L. aureum*–treated groups compared with the H_2_O_2_ group (*p* < 0.01), while the LDH activity and SOD activity of high-dose *L. aureum*–treated groups recovered to no distinct difference levels from the H_2_O_2_ group (*p* < 0.05). In addition, it could be seen in Figure 4D that the MDA level of low-dose *L. aureum*–treated group was significantly lower than the H_2_O_2_ group (*p* < 0.05). However, the MDA levels of middle- and high-dose *L. aureum*–treated groups were significantly increased compared with both H_2_O_2_- and H_2_O_2_-untreated groups (*p* < 0.01).

The aforementioned results indicated that all three doses of *L. aureum* have a protective effect on RAW264.7 cell oxidative damage caused by H_2_O_2_ to a certain degree. Catalase (CAT) is the most significant H_2_O_2_ scavenger enzyme in cells, which plays an important role in the ROS scavenging system of organisms (55). Lactate dehydrogenase (LDH) is an oxidoreductase that catalyzes the reversible conversion between lactate and pyruvate, which exists widely in cells of organisms (56). Superoxide dismutase (SOD) is a superoxide anion scavenger enzyme, which catalyzes the disproportionation of superoxide anions to generate H_2_O_2_ and O_2_. SOD plays an important role in the biological antioxidant system, which is the first line of defense against the damage mediated by reactive oxygen species (ROS) (57). Oxygen free radicals act on the unsaturated fatty acids of lipids to generate lipid peroxide, which is gradually decomposed into a series of compounds including malondialdehyde (MDA). Detecting the level of MDA can reflect the level of cellular lipid oxidation (58). To sum up, all three doses of *L. aureum* could alleviate the oxidative damage of cells, but only low-dose *L. aureum*–treated group have the effect of reducing MDA level. Therefore, *L. aureum* mainly exerted its antioxidant effect by increasing the intracellular activity of CAT, SOD, and LDH.

### Network Pharmacology Analysis

#### Identification of Bioactive Ingredients and Targets

**Figure 5:**
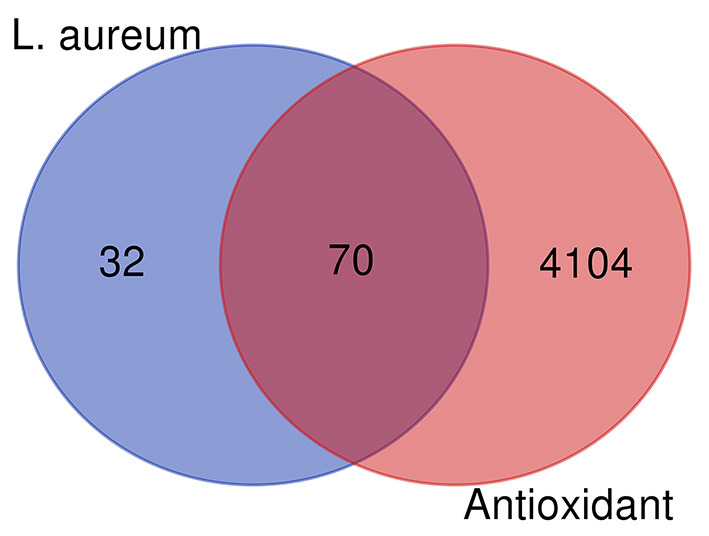
Venn diagram showing all candidates, interaction targets of *L. aureum*, and antioxidant.

The bioactive components in *L. aureum* were determined by literatures and TCMSP database. According to the setting parameters, those where the OB ≥30% and DL ≥0.18, seven bioactive components were identified. In addition, four components with low oral bioavailability were also included considering their reported antioxidant activity or high DL value, such as myricetin (59). The targets of 11 bioactive components (Table 1) were screened and identified through the TCMSP and SwissTargetPrediction databases. A total of 102 targets were obtained after the duplicates were deleted. Furthermore, the 4,174 antioxidant-associated genes were collected through the Genecard database following bioinformatics analysis. After plotting the Venn diagram through the online tool “jvenn” (available online: http://jvenn.toulouse.inra.fr/app/example.html) (60), 70 interaction targets were obtained (Figure 5).

**Table 1:** Information of bioactive components of *L. aureum*.

**Ingredient ID**	**Ingredient**	**OB (%)**	**DL**
MOL001494	Ethyl linoleate	42	0.19
MOL002032	Dioctyl phthalate	40.59	0.4
MOL001973	Sitosterol acetate	40.39	0.85
MOL001494	Ethyl linoleate	42	0.19
MOL005190	Eriodictyol	71.79	0.24
MOL004328	Naringenin	59.29	0.21
MOL000098	Quercetin	46.43	0.28
MOL000422	Kaempferol	41.88	0.24
MOL002008	Myricetin	13.75	0.31
MOL007227	Myricetin-3-O-β-D-glucopyranoside	1.43	0.79
MOL009801	Myricetin-3-O-β-D-galactopyranoside	2.68	0.79
MOL000350	Homoeriodictyol	2.21	0.27

#### Construction of PPI Network and Analysis of Hub Genes

**Figure 6:**
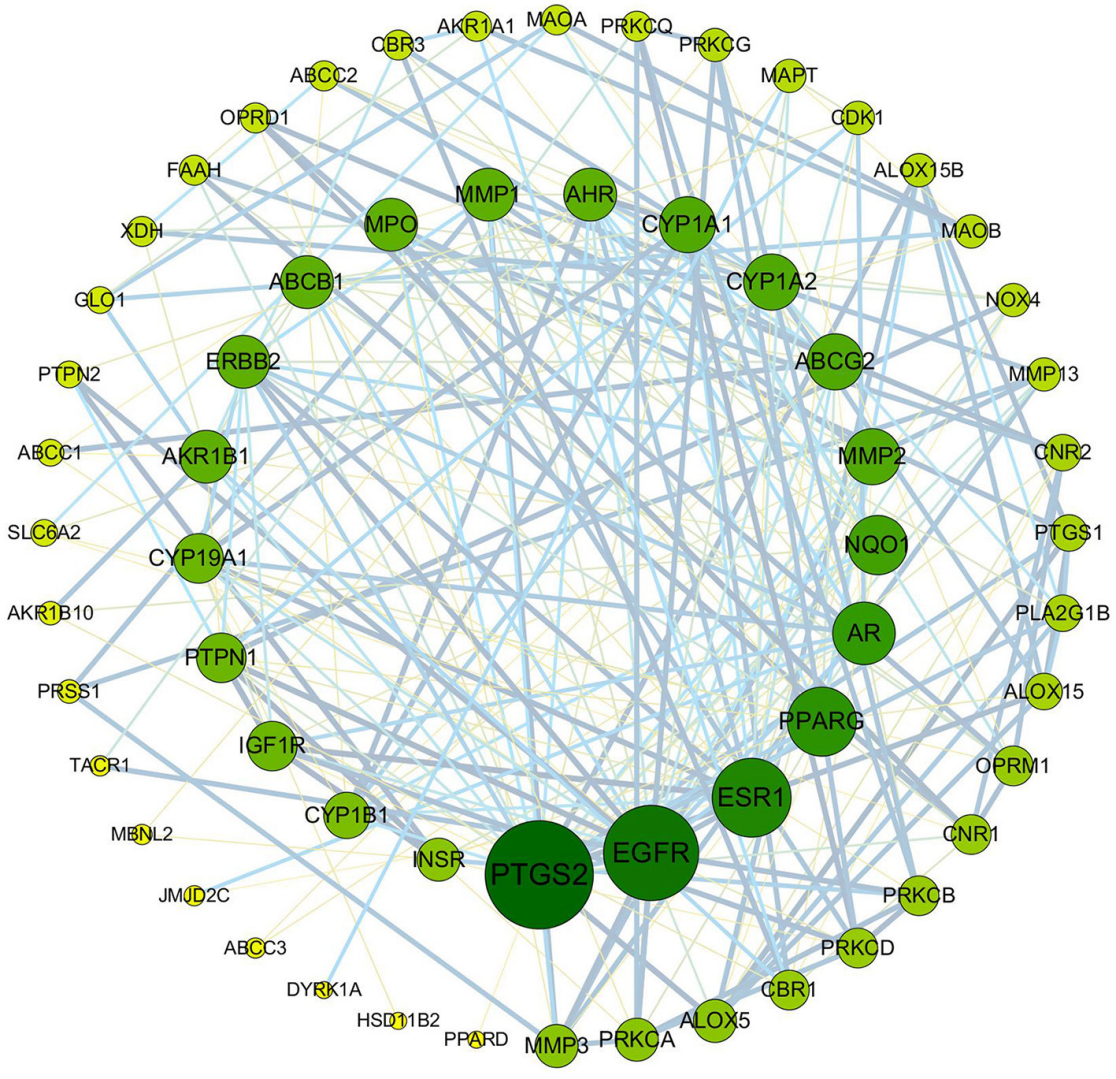
PPI network of anti-oxidative targets of *L. aureum*. The potential targets are represented by the nodes, and the interactions between targets are represented by the edges. The larger degree of targets was indicated by the color from dark to light and the size from big to small. The combined score of targets was indicated by the thickness of lines.

To clarify the potential antioxidant mechanisms of *L. aureum*, the 70 obtained intersection targets were entered into STRING database to obtain the function-related PPI data. The medium confidence interaction score (0.4) was set to construct the PPI network. As shown in Figure 6, the potential targets were represented by the nodes, and the interactions between targets were represented by the edges. The larger degree of targets was indicated by the color from dark to light and the size from big to small. The combined score of targets was indicated by the thickness of lines. It was confirmed by the PPI network analysis results that the lowest combined score between nodes was 0.4, while the highest combined score was 0.999 (Supplementary File 1). According to the degree value, the top 20 hub genes were screened out (Table 2).

**Table 2:** Top 20 hub genes of *L. aureum* antioxidant PPI network.

**No**.	**Target**	**Description**	**Degree**
1	PTGS2	Encoding cyclooxygenase-2	30
2	PPARG	Peroxisome proliferator activated receptor gamma	27
3	ESR1	Estrogen receptor 1	23
4	EGFR	Epidermal growth factor receptor	22
5	ERBB2	Tyrosine kinase receptor 2	16
6	ABCB1	ATP binding cassette subfamily B member 1	4
	CYP1A1	Cytochrome P450 family 1 subfamily A member 1	
	CYP1A2	Cytochrome P450 family 1 subfamily A member 2	
	NQO1	NAD(P)H quinone dehydrogenase 1	
10	ABCG2	ATP binding cassette subfamily G member 2	13
	AR	Androgen receptor	
12	AKR1B1	Aldo-keto reductase family 1 member B	12
	CYP19A1	Cytochrome P450 family 19 subfamily A member 1	
	IGF1R	Insulin-like growth factor 1 receptor	
15	AHR	Aryl hydrocarbon receptor	11
	INSR	Insulin receptor	
	MMP2	Matrix metallopeptidase 2	
	PRKCA	Protein kinase C alpha	
	MPO	Myeloperoxidase	
20	PRKCB	Protein kinase C beta	10
	PTPN1	Protein tyrosine phosphatase non-receptor type 1	

#### GO Biological Functions and KEGG Pathway Enrichment Analyses

**Figure 7:**
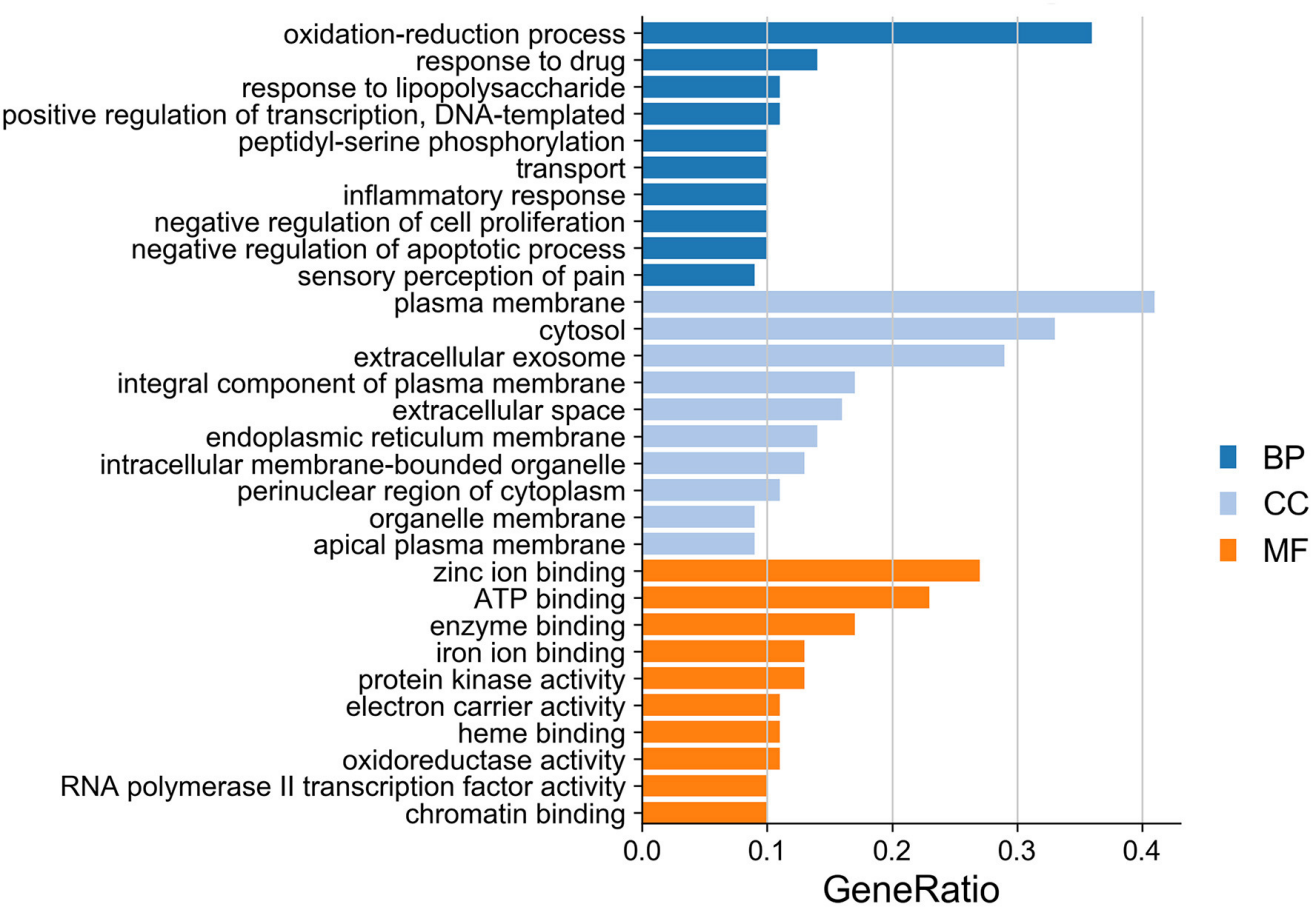
GO enrichment analysis of targets in bar diagram. The top 10 significant enriched terms in biological process (BP), cellular components (CC), and molecular function (MF) are illustrated, respectively.

The ClusterProfiler in R language was used to perform the GO enrichment analysis of the targets in the PPI network. As shown in Figure 7, the biological processes (BP) were mainly involved in the oxidation-reduction process, response to drug, response to lipopolysaccharide, positive regulation of transcription, DNA templating, peptidyl-serine phosphorylation, transport, inflammatory response, negative regulation of cell proliferation, negative regulation of apoptotic process, and sensory perception of pain. The top 10 significant enriched terms in cellular components (CC) included plasma membrane, cytosol, extracellular exosome, integral component of plasma membrane, extracellular space, endoplasmic reticulum membrane, intracellular membrane-bounded organelle, perinuclear region of cytoplasm, organelle membrane, and apical plasma membrane. The molecular function (MF) results suggested that these targets mostly related to zinc ion binding, ATP binding, enzyme binding, iron ion binding, protein kinase activity, electron carrier activity, heme binding, oxidoreductase activity, RNA polymerase II transcription factor activity, ligand-activated sequence-specific DNA binding, and chromatin binding. Moreover, the KEGG pathway enrichment analysis (Table 3) has suggested that target-based KEGG pathways were associated mainly in pathways in cancer, HIF-1 signaling pathway, Rap1 signaling pathway, ErbB signaling pathway, VEGF signaling pathway, mTOR signaling pathway, and PPAR signaling pathway. Among them, HIF-1 signaling pathway (61), ErbB signaling pathway (62), and mTOR signaling pathway (63) were oxidative related pathways.

**Table 3:** KEGG pathway enrichment analysis.

**Term**	**Description**	**Gene ratio**	***P*-value**	**Benjamini**
hsa05200	Pathways in cancer	12/70	3.1E-04	6.5E-03
hsa04066	HIF-1 signaling pathway	7/70	1.3E-04	4.7E-03
hsa04015	Rap1 signaling pathway	7/70	7.5E-03	4.0E-02
hsa04012	ErbB signaling pathway	5/70	5.7E-03	3.6E-02
hsa04014	Ras signaling pathway	7/70	1.1E-02	5.1E-02
hsa04370	VEGF signaling pathway	4/70	1.4E-02	6.3E-02
hsa04150	mTOR signaling pathway	3/70	8.3E-02	2.2E-01
hsa04931	Insulin resistance	6/70	1.9E-03	2.0E-02
hsa04915	Estrogen signaling pathway	5/70	8.9E-03	4.5E-02
hsa03320	PPAR signaling pathway	4/70	1.8E-02	7.2E-02

#### Integrative Network Construction

**Figure 8:**
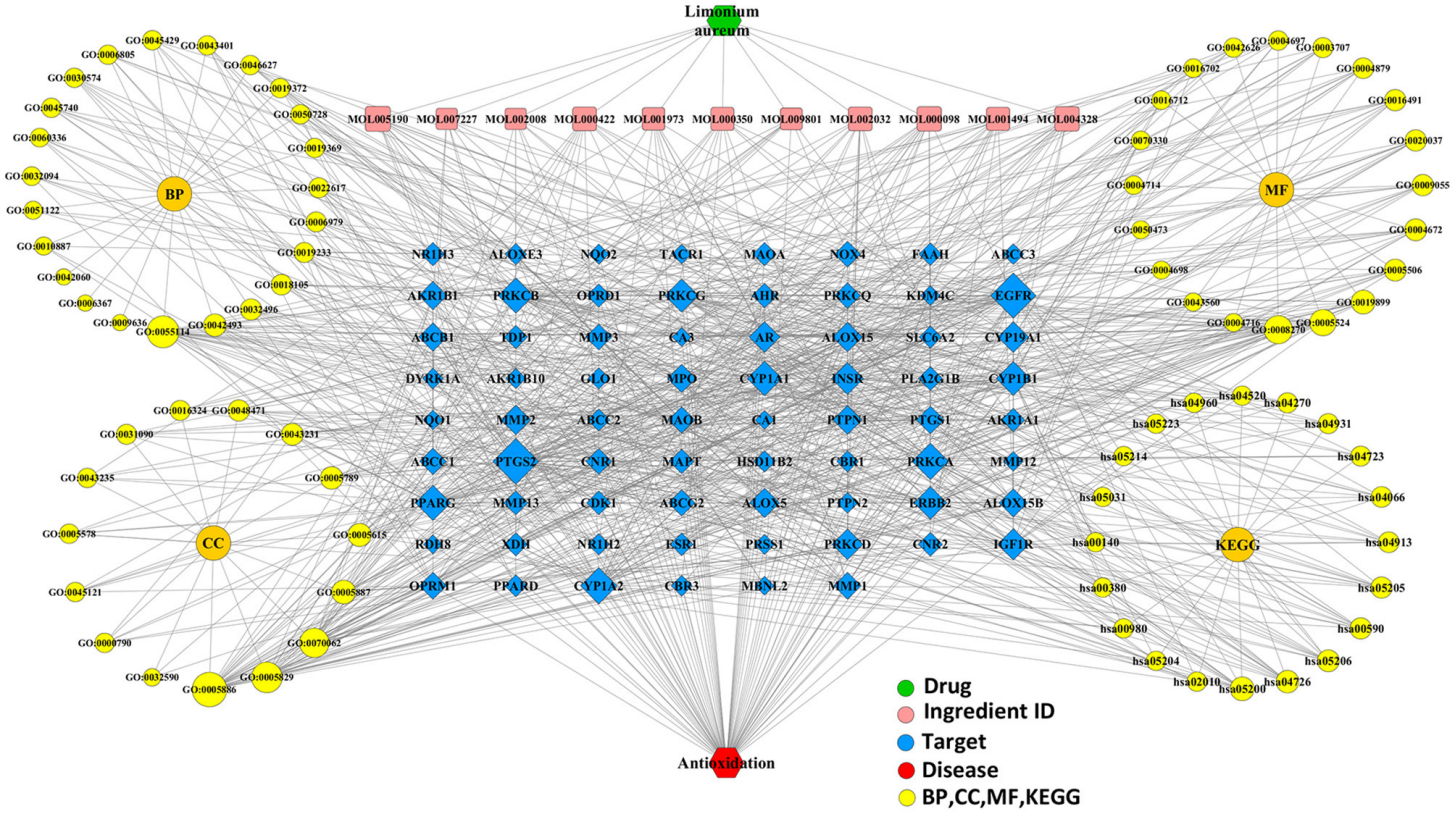
The integrated visualization network based on the network pharmacology findings.

For further analysis, the integrated visualization network (Figure 8) was constructed by using the Cytoscape software, which included interactions of bioactive ingredients of *L. aureum, L. aureum*–affected antioxidant targets, and target-related pathways.

### The Influences of the mRNA Expression of the Hub Genes by *L. aureum*

**Figure 9:**
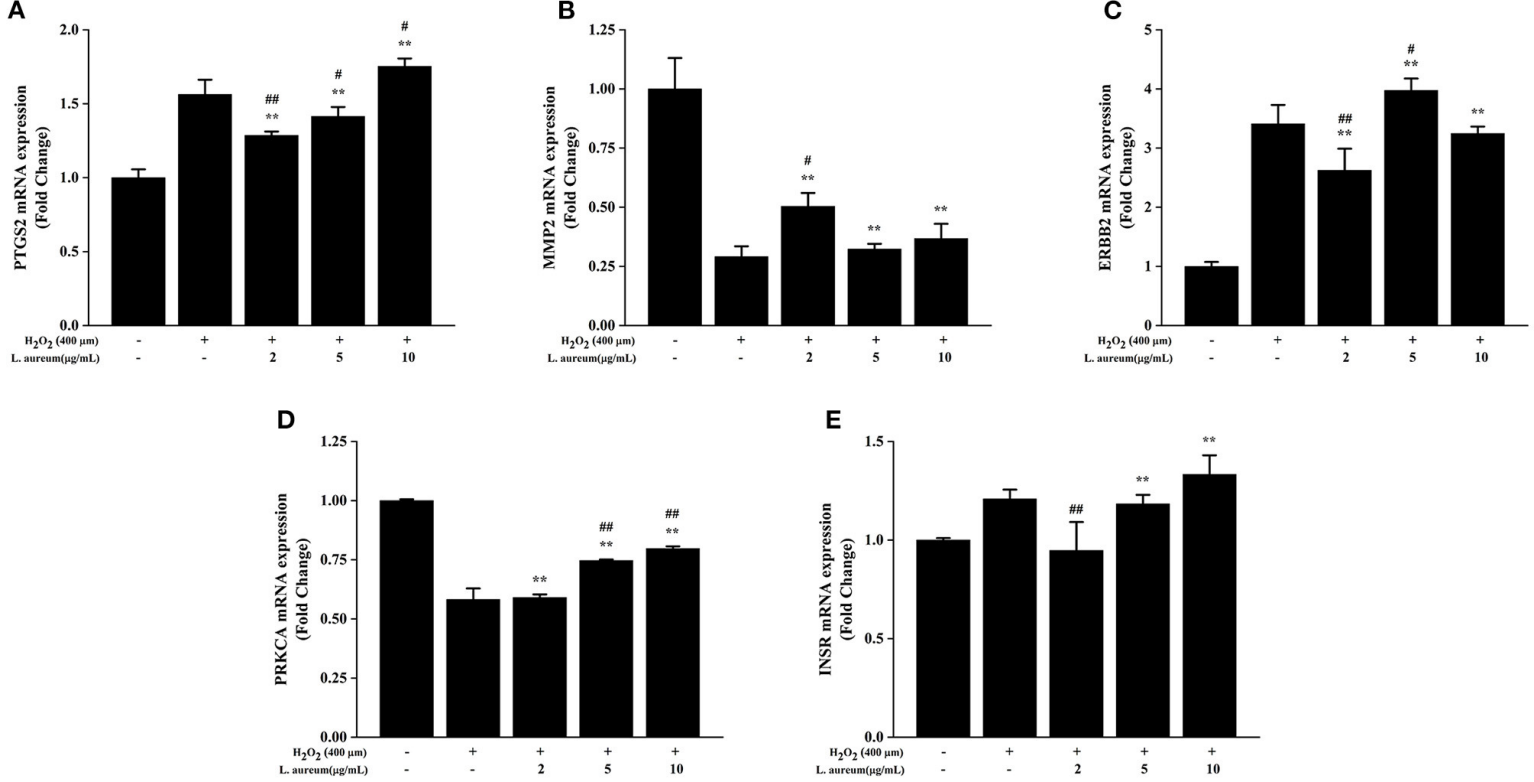
Effects of *L. aureum* on expression levels of key mRNA in RAW264.7 Cells. The expression of **(A)** PTGS2 (COX-2), **(B)** MMP2, **(C)** ERBB2, **(D)** PRKCA, and **(E)** INSR mRNA levels were determined by qRT-PCR. The results are expressed as the mean ± SD (*n* = 3). **p < 0.01 vs. H_2_O_2_-untreated group, #*p* < 0.05 and ##*p* < 0.01 vs. H_2_O_2_ group were considered statistically significant differences.

The effects of *L. aureum* on the hub genes predicted by network pharmacology were investigated through the measurements of mRNA levels of PTGS2 (COX-2), MMP2, ERBB2, PRKCA, and INSR by quantitative real-time PCR. The selection of these five genes was based on their involvement in the enriched oxidative stress–related signaling pathways or a higher combination scores in the PPI network. As demonstrated in Figure 9, the mRNA expression of PTGS2 (COX-2), ERBB2, and INSR was significantly increased after H_2_O_2_ stimulation (400 μM), the upregulation folds were 1.56, 3.41, and 1.21, respectively. Whereas, the mRNA expression of MMP2 and PRKCA was significantly decreased, the downregulation folds were 0.29 and 0.58, respectively. Moreover, compared with the H_2_O_2_-treated group, the expression levels of PTGS2 in low-dose (0.83-fold, *p* < 0.01) and medium-dose (0.91-fold, *p* < 0.05) *L. aureum*–treated groups were significantly downregulated. MMP2 mRNA expression level in the low-dose group was upregulated 1.72 times to H_2_O_2_-treated group (*p* < 0.01), while the upregulation folds of PRKCA mRNA expression level in medium-dose and high-dose groups were 1.29 and 1.38 (*p* < 0.01). In addition, mRNA expression level for ERBB2 in medium-dose group was significantly increased 1.17-fold (*p* < 0.05) and for INSR in low-dose group was significantly decreased 0.79-fold (*p* < 0.01).

## Discussion

The concept of oxidative stress was first proposed by Sies in 1985, which is caused by the imbalance between prooxidation and antioxidation (64). Accompanied with the increased levels of intracellular oxidants, there are two potentially significant influences linked to the development of age-related diseases that included damages of various intracellular components and trigger activation of specific signaling pathways (2). Accumulating evidence implicates that the destruction of redox homeostasis is involved in the processes of many diseases: obesity (65), diabetes (66), aging (67), neurodegenerative diseases (68), cardiovascular disease (69), immunology (70), cancer (71), etc. It has been reported that many traditional medicinal plants have higher antioxidant activity compared with synthetic antioxidants (72). *Limonium aureum* is a traditional medicinal plant mostly used to treat wind heat cold, neuralgia, less menstruation, toothache, etc. by its effects of anti-inflammation and anti-oxidation (18, 21, 23). Therefore, *L. aureum* has great value in development and utilization for the treatment of oxidative stress diseases. As a cutting-edge research field, network pharmacology emphasizes the similar holistic thinking with traditional Chinese medicine which is “network target, multiple ingredients therapeutics” (73). Unlike previous studies, this study was the first time to elucidate the antioxidant mechanism of *L. aureum* via the combination of systematic network pharmacology method and cell biological approaches, hoping to lay the foundation for the further research of *L. aureum*.

In this study, a total of 11 bioactive compounds of *L. aureum* (Table 1) that have potential anti-oxidative effects were retrieved from the TCMSP database and literatures. Among them, the antioxidant activity of components including quercetin, kaempferol, and myricetin had been reported. Quercetin is a naturally occurring flavonoid widely distributed in various plants and foods, which has antioxidant, antiviral, and antibacterial effects. It has been found that quercetin suppresses endothelial cell damage by countering the H_2_O_2_-induced oxidative stress via upregulating the expression of heme oxygenase-1 (HMOX-1) (27). As one of the most common dietary flavonoids, kaempferol has biological and pharmacological effects such as antioxidant, anti-inflammatory, and anti-cancer. According to the report, the generation of reactive oxygen species and mitochondrial membrane potential compromise of porcine oocytes induced by H_2_O_2_ can be prevented by kaempferol. The H_2_O_2_-induced extent of DNA damage and autophagy in blastocysts was also observed to be reduced with the kaempferol supplementation (28). Previous studies have proved that myricetin has a variety of pharmaceutical activities. For instance, myricetin could significantly increase the SOD level and total antioxidant capacity in H_2_O_2_-induced oxidative stress model of bovine mammary epithelial cells (bMECs), while the MDA and ROS levels were decreased (29). The mechanism was found that the H_2_O_2_-induced oxidative stress in bMECs was inhibited by myricetin through AMPK/NRF2 signaling pathway. These findings suggested that *L. aureum* exerts its anti-oxidative effects through multiple ingredients and multiple targets.

To investigate the *in vitro* antioxidant activity of *L. aureum*, four classical assays were selected in this study. Results showed that *L. aureum* have strong DPPH and hydroxyl scavenging activity, ferrous ion-chelating ability, and ferric reducing power. All these *in vitro* antioxidant activities of *L. aureum* were in a concentration-dependent manner. Next, to elucidate further the antioxidant mechanism of *L. aureum*, a PPI network consisting of 51 nodes and 201 interaction edges was constructed. Based on the analysis of topological properties, the hub genes including PTGS-2 (COX-2), MMP2, ERBB2, PRKCA, and INSR were then screened out. H_2_O_2_-induced RAW264.7 cells are the typical oxidative stress model. Therefore, we used them to investigate the *in vivo* antioxidant effects of *L. aureum*. The enzyme activity assay showed that *L. aureum* promotes activities of CAT, LDH, and SOD when the concentration was 2 or 5 μg/ml, while the MDA level assay intuitively showed that *L. aureum* shows the inhibition effect of intracellular MDA level only at 2 μg/ml.

Moreover, quantitative real-time PCR further verified that the mRNA expression levels of PTGS-2 (COX-2), MMP2, ERBB2, PRKCA, and INSR significantly differ between H_2_O_2_-induced group and *L. aureum*–treated group, respectively. Specifically, the mRNA expression levels of PTGS-2 and INSR were significantly downregulated with the low-dose *L. aureum*–treated group, and the downregulation folds were 0.83 and 0.79, respectively. At the same time, the upregulation folds of mRNA expression level for MMP2 in low-dose group and ERBB2 in medium-dose group were 1.72 and 1.17, respectively. Besides, mRNA expression levels for PRKCA with medium-dose and high-dose *L. aureum* treatment were significantly upregulated 1.29- and 1.38-fold. The aforementioned results suggested that these predicted core genes play important roles in the process of *L. aureum* exerting its antioxidant effects. PTGS-2 (encoding cyclooxygenase-2, COX-2) is a rate-limiting enzyme of the production of prostaglandin metabolites, and its expression can be upregulated by reactive oxygen intermediates generated by oxidative stress (74, 75). It has been suggested that glutathione (GSH), which reduces prostaglandin G2 (PGG2) to prostaglandin H2 (PGH2), is depleted due to the PTGS-2 (COX-2) activity, leading to the decreasing of cells reducing power (76). As a member of the epidermal growth factor receptor (EGFR) family, tyrosine kinase receptor 2 (ERBB2) is important to the research of cancer biology and cardiac function and development. It has been suggested by experimental evidences that ERBB2 was involved in the regulation of antioxidant defenses in cancer and the protection against cardiomyocyte oxidative stress and death, which was due to pathways that converged on preventing oxidative stress and induced further activation and upregulation by the upregulation of ERBB2 (77).

PRKCA (protein kinase C alpha) belongs to the protein kinase C (PKC) family, which is a family of serine/threonine-specific protein kinases. They are involved downstream of almost all membrane-related signal transduction pathways, and their activations require Ca^2+^ and diacylglycerol (DAG) (78, 79). A variety of oxidative stress–related diseases have been found to be affected by the expression of PRKCA. As a cardiac contractile node integrator, PRKCA was suggested to profoundly affect the propensity of heart failure through intracellular Ca^2+^ sensing and signal transduction events (78). An observation showed that PRKCA mRNA levels in blood of multiple sclerosis patients were significantly lower, which has been consistent with the results of their experiments that higher levels of PRKCA expression conferred by alleles were related to the protective signal (79). It also had been noted that PRKCA has a regulation effect of NF-κB-induced IL-1 expression in HepG2 cells (80) and LPS-induced IL-1 and iNOS expression in RAW264.7 cells (81). Furthermore, the PTGS2 (COX-2) expression induced by LPS in RAW264.7 cells was strongly inhibited by the overexpression of dominant-negative mutant of PRKCA (81).

The insulin receptor (INSR) belongs to tyrosine kinase family, which is a member of the ligand-activated receptor of transmembrane signal proteins. As a fundamentally important regulator, it is associated with the differentiation, growth, and metabolism of cell (82). The binding of insulin under normal state to INSR allowed the performing of insulin functions, which resulted in the activation of downstream signaling cascades and the autophosphorylation of INSR (83). Literature indicated that the antioxidant curcumin disrupted insulin signaling in hepatic stellate cells (HSCs) by inhibiting the gene expression of INSR and reducing the phosphorylation level of INSR (84). Besides, the insulin-induced oxidative stress in HSCs was attenuated by curcumin through its induction of gene expression of glutamate cysteine ligase, enabled *de novo* synthesis of glutathione, and inhibited the gene expression of INSR. The aforementioned results of studies were similar to the present study. However, matrix metalloproteinases (MMPs), which are a zinc-dependent family of endopeptidases, are responsible for the regulation of numerous protein activities in many pathological conditions, especially MMP2 and MMP9 (85). MMP2 was suggested to play a key role in the reduction of nicotinamide adenine dinucleotide phosphate oxidase (NOX2) activity and the eventual formation of ROS (86). Contrary to other studies (85, 86), our study found that the expression of MMP2 was downregulated in the positive control group. The possible reason might be that the activation of MMPs could not be induced by one-time treatment with H_2_O_2_ (87), or it might be caused by the different characteristics of respective studies.

In addition, the antioxidant effect of *L. aureum* was mainly enriched in the HIF-1 signaling pathway and ERBB signaling pathway demonstrated by the KEGG enrichment analysis. Hypoxia-inducible factor 1 (HIF-1) is a hypoxia-related protective transcription factor, which mainly regulates a variety of hypoxia-inducible genes under hypoxia. It was induced by stimulants (nitric oxide or various growth factors) and the reduction of oxygen availability. Evidences were provided to illustrate the mitochondrial stress reduction effect of HIF-1, which was caused by the inhibition of mitochondrial fission, improvement of mitochondrial oxygen metabolism, neutralization of ROS, and regulation of inflammatory response. Cell apoptosis was promoted when the HIF-1 signaling pathway in epidermal HaCaT cells under oxidative stress microenvironment was inhibited by inverted formin-2 (INF2) (61). It has also been reported that the regulation of HIF-1a expression was involved in reactive oxygen species and Nrf2 signaling. Knockdown of Nrf2 or elimination of ROS impaired the activation of HIF-1 signaling pathway, thereby attenuating the binding of HIF-1 to the VEGF promoter, which was induced by follicle-stimulating hormone (FSH) (88). Furthermore, intracellular signaling pathways and extracellular growth factor ligands are bound through the ERBB family, which is a member of receptor tyrosine kinases (RTKs), to regulate various biological reactions. The common target downstream of all ERBB receptors is the mitogen-activated protein kinase (MAPK) pathway which is activated by SHC or GRB2, while most ERBBs directly or indirectly activate the phosphatidylinositol-3-kinase (PI3K) pathway. It has been demonstrated that neuregulin-1 beta (NRG) attenuated doxorubicin-induced oxidative stress in rat ventricular cardiomyocytes. Trastuzumab, an antibody targeting the ERBB2 receptor, blocked this beneficial effect of NRG by inhibiting the ERBB2/NRG signaling pathway (89). The consistent conclusion was reported that the ERBB and its downstream AKT/PI3K signaling could be activated by NRG1 to improve mitochondrial function and exert the antioxidant effect (62).

**Figure 10:**
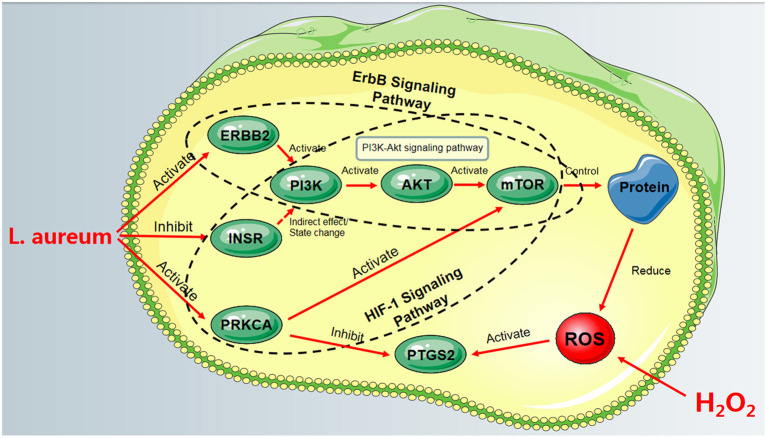
The process of *L. aureum* mitigating oxidative stress of cells.

Based on the aforementioned information, PRKCA, INSR, and ERBB2 could regulate their common downstream PI3K/AKT signaling pathway, which was the oxidative stress-related pathway. These pathways were contained in HIF-1 signaling pathway and ErbB signaling pathway, respectively. Meanwhile, the activation of PRKCA in the HIF-1 signaling pathway could inhibit the expression of PTGS2. These results suggested that HIF-1 and ErbB signaling pathways may be the key pathways for *L. aureum* to exert its antioxidant effect. The fact that *L. aureum* can regulate the expression of these hub genes is in accordance with the predicted results. To sum up, the regulation of ErbB and HIF-1 signaling pathways by regulating the expression of PRKCA, INSR, ERBB2, and PTGS2 may be the reason for its mitigation of oxidative damage of cells (Figure 10).

## Conclusion

Our studies combined the systematic network pharmacology method with cell biological approaches to elucidate the underlying molecular mechanism of *L. aureum* on oxidative stress. From open source databases, a total of 11 bioactive compounds, 102 *L. aureum*–related targets, and 70 interaction targets of *L. aureum* and antioxidant were obtained. Moreover, the hub genes including PTGS2 (COX-2), MMP2, ERBB2, PRKCA, and INSR were screened out based on the analysis of the topological character of the protein–protein interaction network. Then, the mRNA expression of these hub genes was verified by performing the experimental *in vitro* validation. This finding lays a foundation for further elucidating the anti-oxidative damage mechanism of *L. aureum*. Considering the complexity of the oxidative stress process and the deficiencies in network prediction, further research is necessary to confirm our findings.

## Data Availability Statement

The original contributions presented in the study are included in the article/Supplementary Material, further inquiries can be directed to the corresponding author/s.

## Author Contributions

ZY, YM, and FC conceived and designed the study. ZY and YM conducted the experiments. ZY drafted the article. YL and BH supervised the study. HZ, RS, XW, and JL reviewed the methods and the results. All authors contributed to the article and approved the submitted version.

## Funding

This work was supported by grants from the Gansu Province Science and Technology Foundation Program for Youths (No. 21JR7RA034), the Chinese Academy of Agricultural Sciences Innovative Project Veterinary Natural Medicine (No. 25-LZIHPS-03), Lanzhou Institute of Animal Husbandry and Veterinary Medicine of CAAS Fundamental Research Funds (No. 1610322021006), and Construction Project of Jinan (No. 00252019025).

## Conflict of Interest

The authors declare that the research was conducted in the absence of any commercial or financial relationships that could be construed as a potential conflict of interest.

## Publisher's Note

All claims expressed in this article are solely those of the authors and do not necessarily represent those of their affiliated organizations, or those of the publisher, the editors and the reviewers. Any product that may be evaluated in this article, or claim that may be made by its manufacturer, is not guaranteed or endorsed by the publisher.

## Supplementary Material

Supplementary Table S1 The qRT-PCR primers for the hub genes.

Supplementary Figure S1 The effects of *L. aureum* and hydrogen peroxide on the viability of RAW264.7 cells. **(A)** The effect of hydrogen peroxide on the cell viability. Cells were treated with different concentrations of hydrogen peroxide (0, 100, 200, 400, 500, 600, 800 qM) for 24 h. **(B)** The effect of *L. aureum* on the cell viability. Cells were treated with different concentrations of *L. aureum* (0, 1, 2, 5, 8, 10, 20, 40 qg/ml) for 24 h. The results were expressed as the mean + standard deviation of three independent experiments. ^*^*P* < 0.05 and ^**^*P* < 0.01 compared with hydrogen peroxide-untreated/*L. aureum*-untreated cells was considered statistically significant differences.

Supplementary File 1 The PPI network analysis results.

## References

[1] Ungvari Z Bagi Z Feher A Recchia FA Sonntag WE Pearson K. Resveratrol confers endothelial protection via activation of the antioxidant transcription factor Nrf2. Am J Physiol Heart C. (2010) 299:H18–24. 10.1152/ajpheart.00260.2010 20418481 PMC2904129

[2] Finkel T Holbrook NJ. Oxidants, oxidative stress and the biology of ageing. Nature. (2000) 408:239–47. 10.1038/35041687 11089981

[3] Sahreen S Khan MR Khan RA. Evaluation of antioxidant activities of various solvent extracts of Carissa opaca fruits. Food Chem. (2010) 122:1205–11. 10.1016/j.foodchem.2010.03.120 PMC5561747 29086845

[4] Zhu Q Zeng J Li J Chen XM Miao JX Jin QY. Effects of compound Centella on oxidative stress and Keap1-Nrf2-ARE pathway expression in diabetic kidney disease rats. Evid Based Compl Alt. (2020) 2020:1–13. 10.1155/2020/9817932 32595756 PMC7277064

[5] Wu WL Papagiannakopoulos T. The pleiotropic role of the Keap1/Nrf2 pathway in cancer. Annu Rev Cancer Biol. (2020) 4:413–35. 10.1146/annurev-cancerbio-030518-055627

[6] Guo Z Mo Z. Keap1-Nrf2 signaling pathway in angiogenesis and vascular diseases. J Tissue Eng Regen M. (2020) 14:869–83. 10.1002/term.3053 32336035

[7] Zhang Y Wang G Wang T Cao W Zhang LX Chen XY. Nrf2-Keap1 pathway-mediated effects of resveratrol on oxidative stress and apoptosis in hydrogen peroxide-treated rheumatoid arthritis fibroblast-like synoviocytes. Ann NY Acad Sci. (2019) 1457:166–78. 10.1111/nyas.14196 31475364

[8] Teleanu RI Chircov C Grumezescu AM Volceanov A Teleanu DM. Antioxidant therapies for neuroprotection - a review. J Clin Med. (2019) 8:1659. 10.3390/jcm8101659 31614572 PMC6832623

[9] Hopkins AL. Network pharmacology: the next paradigm in drug discovery. Nat Chem Biol. (2008) 4:682–90. 10.1038/nchembio.118 18936753

[10] Hopkins AL. Network pharmacology. Nat Biotechnol. (2007) 25:1110–1. 10.1038/nbt1007-1110 17921993

[11] Csermely P Korcsmaros T Kiss HJM London G Nussinov R. Structure and dynamics of molecular networks: a novel paradigm of drug discovery. A comprehensive review. Pharmacol Therap. (2012) 138:333–408. 10.1016/j.pharmthera.2013.01.016 23384594 PMC3647006

[12] Law V Knox C Djoumbou Y Jewison T Guo AC Liu YF. DrugBank 40: shedding new light on drug metabolism. Nucleic Acids Res. (2014) 42:1091–7. 10.1093/nar/gkt1068 24203711 PMC3965102

[13] Li R Ma XY Song YQ Zhang YY Xiong WB Li L Zhou LM. Anti-colorectal cancer targets of resveratrol and biological molecular mechanism: Analyses of network pharmacology, human and experimental data. J Cell Biochem. (2019) 120:11265–73. 10.1002/jcb.28404 30719773

[14] Li S Zhang B. Traditional Chinese medicine network pharmacology: theory, methodology and application. Chin J Nat Med. (2013) 11:110–20. 10.1016/S1875-5364(13)60037-0 23787177

[15] Li S. Network Pharmacology. Singapore: Springer Press (2021). p. 432–6.

[16] Li YQ Chen Y Fang JY Jiang SQ Li P Li F. Integrated network pharmacology and zebrafish model to investigate dual-effects components of *Cistanche tubulosa* for treating both Osteoporosis and Alzheimer's Disease. J Ethnopharmacol. (2020) 254:112764. 10.1016/j.jep.2020.112764 32173426

[17] Zhang R Zhu X Bai H Ning K. Network pharmacology databases for traditional Chinese medicine: review and assessment. Front Pharmacol. (2019) 10:123. 10.3389/fphar.2019.00123 30846939 PMC6393382

[18] Jiangsu New Medicine. The Dictionary of Traditional Chinese Medicine. Shanghai: The People Press of Shanghai (1977).

[19] Liu SW. Flora of Qinghai. Xining: The People Press of Qinghai (1997).

[20] Yang YC. Tibetan Medicine. Xining: The People Press of Qinghai (1991).

[21] Ye G Huang C. Flavonoids of *Limonium aureum*. Chem Nat Compd^+^. (2006) 42:232–4. 10.1007/s10600-006-0089-3

[22] Liu XF Qian JL Lin P Xue HY Yang L. Study on *Limonium aureum* endophytic fungi and its antibacterial activity. Adv. Mat. Res. (2013) 634–8:1071–5. 10.4028/www.scientific.net/AMR.634-638.1071

[23] Geng DD Chi XF Dong Q Hu FZ. Antioxidants screening in *Limonium aureum* by optimized on-line HPLC–DPPH assay. Ind Crops Prod. (2015) 67:492–7. 10.1016/j.indcrop.2015.01.063

[24] Liu Y Shang RF Cheng FS Wang XH Hao BC Liang JP. Flavonoids and phenolics from the flowers of *Limonium aureum*. Chem Nat Compd^+^. (2016) 52:130–1. 10.1007/s10600-016-1568-9

[25] Zhang SL Ni XL Arif M Zheng J Stubbs A Li CX. NaCl improved Cd tolerance of the euhalophyte Suaeda glauca but not the recretohalophyte *Limonium aureum*. Plant Soil. (2020) 449:303–18. 10.1007/s11104-020-04475-7

[26] Zhou XW Liu ZL Kou L Wu RZ Liu YQ. Chemical constituents of *Limonium aureum* (L.) Hill. J Lanzhou Univ. (2013) 49: 569-572. 10.13885/j.issn.0455-2059.2013.04.013

[27] Tian R Yang ZY Lu NH Peng YY. Quercetin, but not rutin, attenuated hydrogen peroxide-induced cell damage via heme oxygenase-1 induction in endothelial cells. Arch Biochem Biophys. (2019) 676:108157. 10.1016/j.abb.2019.108157 31644887

[28] Yao XR Jiang H Nanxu Y Piao XJ Gao QS Kim N. Kaempferol attenuates mitochondrial dysfunction and oxidative stress induced by H[[sb]]2[[/s]]O[[sb]]2[[/s]] during porcine embryonic development. Theriogenology. (2019) 135:174–80. 10.1016/j.theriogenology.2019.06.013 31226607

[29] Kan XC Liu JX Chen YS Guo WJ Xu DW Cheng J. Myricetin protects against H2O2-induced oxidative damage and apoptosis in bovine mammary epithelial cells. J Cell Physiol. (2020) 236:2684–95. 10.1002/jcp.30035 32885418

[30] Mau JL Lin HC Song SF. Antioxidant properties of several medicinal mushrooms. Food Res Int. (2002) 35:519–26. 10.1016/S0963-9969(01)00150-8 12358482

[31] Ye DY Jiang ZB Zheng FC Wang HM Zhang YM Gao FF. Optimized extraction of polysaccharides from Grateloupia livida (Harv) Yamada and biological activities. Molecules. (2015) 20:16817–32. 10.3390/molecules200916817 26389874 PMC6332504

[32] Wang T Jonsdottir R Olafsdottir G. Total phenolic compounds, radical scavenging and metal chelation of extracts from Icelandic seaweeds. Food Chem. (2009) 116:240–8. 10.1016/j.foodchem.2009.02.041

[33] Katanic J Mihailović V Stanković N Boroja T Mladenović M Solujić S. Dropwort (Filipendula hexapetala Gilib): potential role as antioxidant and antimicrobial agent. Excli J. (2015) 14:1–20. 10.17179/excli2014-479 26417349 PMC4553886

[34] Zhou TY Xiang XW Du M Zhang LF Cheng NX Liu XL. Protective effect of polysaccharides of sea cucumber Acaudina leucoprocta on hydrogen peroxide-induced oxidative injury in RAW2647 cells. Int J Biol Macromol. (2019) 139:1133–40. 10.1016/j.ijbiomac.2019.08.092 31419551

[35] Ge L Cheng K Han J. A network pharmacology approach for uncovering the osteogenic mechanisms of *Psoralea corylifolia* Linn. Evid Based Compl Alt. (2019) 2019:2160175. 10.1155/2019/2160175 31781261 PMC6874874

[36] Ru JL Li P Wang JN Zhou W Li BH Huang C. TCMSP: a database of systems pharmacology for drug discovery from herbal medicines. J Cheminformatics. (2014) 6:1–6. 10.1186/1758-2946-6-13 24735618 PMC4001360

[37] Kim S Chen J Cheng TJ Gindulyte A He J He SQ. PubChem 2019 update: improved access to chemical data. Nucleic Acids Res. (2019) 47:D1102–9. 10.1093/nar/gky1033 30371825 PMC6324075

[38] Daina A Michielin O Zoete V. SwissTargetPrediction: updated data and new features for efficient prediction of protein targets of small molecules. Nucleic Acids Res. (2019) 47:357–64. 10.1093/nar/gkz382 31106366 PMC6602486

[39] Uniprot Consortium. UniProt: a worldwide hub of protein knowledge. Nucleic Acids Res. (2019) 47:D506–15. 10.1093/nar/gky1049 30395287 PMC6323992

[40] Lin YX Shen CQ Wang FJ Fang ZH Shen GM. Network pharmacology and molecular docking study on the potential mechanism of yi-qi-huo-xue-tong-luo formula in treating diabetic peripheral neuropathy. J Diabetes Res. (2021) 2021:9941791. 10.1155/2021/9941791 34159207 PMC8188603

[41] Stelzer G Rosen N Plaschkes I Zimmerman S Twik M Fishilevich S. The GeneCards suite: from gene data mining to disease genome sequence analyses. Curr Protoc Bioinformatics. (2016) S54:1.30.1. 10.1002/cpbi.5 27322403

[42] Chen B Hua ZY Qin XN Li ZJ. Integrated microarray to identify the hub miRNAs and constructed miRNA-mRNA network in neuroblastoma via bioinformatics analysis. Neurochem Res. (2021) 46:1–16. 10.1007/s11064-020-03155-3 33104965

[43] Wu K Wei P Liu M Liang X Su M. To reveal pharmacological targets and molecular mechanisms of curcumol against interstitial cystitis. J Adv Res. (2019) 20:43–50. 10.1016/j.jare.2019.05.003 31193808 PMC6543129

[44] Shannon P Markiel A Ozier O Baliga NS Wang JT Ramage D. Cytoscape: a software environment for integrated models of biomolecular interaction networks. Genome Res. (2013) 13:2498–504. 10.1101/gr.1239303 14597658 PMC403769

[45] Szklarczyk D Gable AL Lyon D Junge A Wyder S Huerta-Cepas J. STRING v11: protein-protein association networks with increased coverage, supporting functional discovery in genome-wide experimental datasets. Nucleic Acids Res. (2019) 47:D607–13. 10.1093/nar/gky1131 30476243 PMC6323986

[46] Tian H Wei LL Yao YX Zeng ZQ Liang X Zhu H. Analysis of the anti-inflammatory and analgesic mechanism of shiyifang vinum based on network pharmacology. Evid Based Compl Alt. (2021) 2021:1–10. 10.1155/2021/8871276 33519947 PMC7817263

[47] Yu GC Wang LG Han YY He QY. clusterProfiler: an R package for comparing biological themes among gene clusters. Omic. (2012) 16:284–7. 10.1089/omi.2011.0118 22455463 PMC3339379

[48] Huang DW Sherman BT Lempicki RA. Systematic and integrative analysis of large gene lists using DAVID bioinformatics resources. Nat Protoc. (2009) 4:44–57. 10.1038/nprot.2008.211 19131956

[49] Rezaei-Sadabady R Eidi A Zarghami N Barzegar A. Intracellular ROS protection efficiency and free radical-scavenging activity of quercetin and quercetin-encapsulated liposomes. Artif Cell Nanomed B. (2016) 44:128–34. 10.3109/21691401.2014.926456 24959911

[50] Xiong QP Li X Zhou RZ Hao HR Li SL Jing Y Zhu C Zhang QH. Extraction, characterization and antioxidant activities of polysaccharides from *E. corneum gigeriae galli*. Carbohydr Polym. (2014) 108:247–56. 10.1016/j.carbpol.2014.02.068 24751271

[51] Tran DL Thi PL Thi TTH Park KD. Novel enzymatically crosslinked chitosan hydrogels with free-radical-scavenging property and promoted cellular behaviors under hyperglycemia. Prog Nat Sci Mater. (2020) 30:661–8. 10.1016/j.pnsc.2020.08.004

[52] Kim JS. Antioxidant activity of Maillard reaction products derived from aqueous and ethanolic glucose-glycine and its oligomer solutions. Food Sci Biotechnol. (2013) 22:39–46. 10.1007/s10068-013-0006-z

[53] Kim JH Nam SH Rico CW Kang MY A. comparative study on the antioxidative and anti-allergic activities of fresh and aged black garlic extracts. Int J Food Sci Tech. (2012) 47:1176–82. 10.1111/j.1365-2621.2012.02957.x

[54] Mokrani A. Madani K. Effect of solvent, time and temperature on the extraction of phenolic compounds and antioxidant capacity of peach (*Prunus persica L*) fruit. Sep Purif Technol. (2016) 162:68–76. 10.1016/j.seppur.2016.01.043

[55] Sellaththurai S Priyathilaka TT Lee J. Molecular cloning, characterization, and expression level analysis of a marine teleost homolog of catalase from big belly seahorse (*Hippocampus abdominalis*). Fish Shellfish Immun. (2019) 89:647–59. 10.1016/j.fsi.2019.03.064 30936047

[56] Khan AA Allemailem KS Alhumaydhi FA Gowder SJT Rahmani AH. The biochemical and clinical perspectives of lactate dehydrogenase: an enzyme of active metabolism. Endocrine. (2020) 20:855–68. 10.2174/1871530320666191230141110 31886754

[57] Zhao HQ Zhang RF Yan XY Fan KL. Superoxide dismutase nanozymes: an emerging star for anti-oxidation. J Mater Chem B. (2021) 9:6939–57. 10.1039/D1TB00720C 34161407

[58] Jiang J Zhuang JY Fan YY Shen B. Mapping of QTLs for leaf malondialdehyde content associated with stress tolerance in rice. Rice Sci. (2009) 16:72–4. 10.1016/S1672-6308(08)60059-1

[59] Song X Tan L Wang M Ren CX Guo CJ Yang B. Myricetin: a review of the most recent research. Biomed Pharmacother. (2021) 134:111017. 10.1016/j.biopha.2020.111017 33338751

[60] Bardou P Mariette J Escudié F Djemiel C Klopp C. jvenn: an interactive Venn diagram viewer. BMC Bioinformatics. (2014) 15:293. 10.1186/1471-2105-15-293 25176396 PMC4261873

[61] Chen ZX Wang CY Yu NZ Si LB Zhu L Zeng A Liu ZF. INF2 regulates oxidative stress-induced apoptosis in epidermal HaCaT cells by modulating the HIF1 signaling pathway. Biomed Pharmacother. (2019) 111:151–61. 10.1016/j.biopha.2018.12.046 30579254

[62] Chang X Lu K Wang L Lv M Fu WJ. Astraglaus polysaccharide protects diabetic cardiomyopathy by activating NRG1/ErbB pathway. Biosci Trends. (2018) 12:149–56. 10.5582/bst.2018.01027 29607874

[63] Sun Y Dai ST Tao J Li YL He Z Liu Q. Taurine suppresses ROS-dependent autophagy via activating Akt/mTOR signaling pathway in calcium oxalate crystals-induced renal tubular epithelial cell injury. Aging. (2020) 12:17353–66. 10.18632/aging.103730 32931452 PMC7521519

[64] Sies H. Oxidative Stress: Introductory Remarks. London: Academic Press (1985). 10.1016/B978-0-12-642760-8.50005-3

[65] Furukawa S Fujita T Shimabukuro M Iwaki M Yamada Y Nakajima Y. Increased oxidative stress in obesity and its impact on metabolic syndrome. J Clin Invest. (2004) 114:1752–61. 10.1172/JCI21625 15599400 PMC535065

[66] Giacco F Brownlee M. Oxidative stress and diabetic complications. Circ Res. (2010) 107:1058–70. 10.1161/CIRCRESAHA.110.223545 21030723 PMC2996922

[67] Xu Z Wei F Shen Q Yu NN Yu K Wang SJ Chen ZG. Rhizoma coptidis and berberine as a natural drug to combat aging and aging-related diseases via anti-oxidation and AMPK activation. Aging Dis. (2017) 8:760–77. 10.14336/AD.2016.0620 29344415 PMC5758350

[68] Silvestro S Sindona C Bramanti P Mazzon E A. state of the art of antioxidant properties of curcuminoids in neurodegenerative diseases. Int J Mol Sci. (2021) 22:3168. 10.3390/ijms22063168 33804658 PMC8003642

[69] Santos J Quadros A Weschenfelder C Garofallo S Marcadenti A. Oxidative stress biomarkers, nut-related antioxidants, and cardiovascular disease. Nutrients. (2020) 12:682. 10.3390/nu12030682 32138220 PMC7146201

[70] Nathan C Cunningham-Bussel A. Beyond oxidative stress: an immunologist's guide to reactive oxygen species. Nat Rev Immunol. (2013) 13:349–61. 10.1038/nri3423 23618831 PMC4250048

[71] Katerji M Filippova M Wongworawat YC Siddighi S Bashkirova S Duerksen-Hughes PJ. Oxidative stress markers in patient-derived non-cancerous cervical tissues and cells. Sci Rep. (2020) 10:19044. 10.1038/s41598-020-76159-2 33149215 PMC7642372

[72] Krishnaiah D Sarbatly R Nithyanandam R. A review of the antioxidant potential of medicinal plant species. Food Bioprod Process. (2011) 89:217–33. 10.1016/j.fbp.2010.04.008

[73] Li S Fan TP Jia W Lu AP Zhang WD. Network pharmacology in traditional Chinese medicine. Evid Based Compl Alt. (2014) 2014:1–2. 10.1155/2014/138460 24707305 PMC3953584

[74] Simmler C Antheaume C Lobstein A. Antioxidant biomarkers from Vanda coerulea stems reduce irradiated HaCaT PGE-2 production as a result of COX-2 inhibition. PLoS ONE. (2016) 5:e13713. 10.1371/journal.pone.0013713 21060890 PMC2965657

[75] Burnett BP Bitto A Altavilla D Squadrito F Levy RM Pillai L. Flavocoxid inhibits phospholipase A2, peroxidase moieties of the cyclooxygenases (COX), and 5-lipoxygenase, modifies COX-2 gene expression, and acts as an antioxidant. Mediat Inflamm. (2011) 2011:385780. 10.1155/2011/385780 21765617 PMC3134205

[76] Laube M Kniess T Pietzsch J. Development of antioxidant COX-2 inhibitors as radioprotective agents for radiation therapy — a hypothesis - driven review. Antioxidants. (2016) 5:14. 10.3390/antiox5020014 27104573 PMC4931535

[77] Belmonte F Das S Sysa-Shah P Sivakumaran V Stanley B Guo X. ErbB2 overexpression upregulates antioxidant enzymes, reduces basal levels of reactive oxygen species, and protects against doxorubicin cardiotoxicity. Am J Physiol Heart C. (2015) 309:H1271–80. 10.1152/ajpheart.00517.2014 26254336 PMC4666964

[78] Braz JC Gregory K Pathak A Zhao W Sahin B Klevitsky R. PKC-alpha regulates cardiac contractility and propensity toward heart failure. Nat Med. (2004) 10:248–54. 10.1038/nm1000 14966518

[79] Paraboschi EM Rimoldi V Solda G Tabaglio T. Dall'Osso C Saba E. Functional variations modulating PRKCA expression and alternative splicing predispose to multiple sclerosis. Hum Mol Genet. (2014) 23:6746–61. 10.1093/hmg/ddu392 25080502

[80] Kim SJ Chun JS. Protein kinase C alpha and zeta regulate nitric oxide-induced NF-kappa B activation that mediates cyclooxygenase-2 expression and apoptosis but not dedifferentiation in articular chondrocytes. Biochem Biophys Res Commun. (2003) 303:206–11. 10.1016/S0006-291X(03)00305-X 12646188

[81] Giroux M Descoteaux A. Cyclooxygenase-2 expression in macrophages: modulation by protein kinase C-alpha. J Immunol. (2000) 165:3985–91. 10.4049/jimmunol.165.7.3985 11034408

[82] Lee J Pilch PF. The insulin receptor: structure, function, and signaling. Am J Physiol. (1994) 266:C319–34. 10.1152/ajpcell.1994.266.2.C319 8141246

[83] Khamzina L Gruppuso PA Wands JR. Insulin signaling through insulin receptor substrate 1 and 2 in normal liver development. Gastroenterology. (2003) 125:572–85. 10.1016/S0016-5085(03)00893-X 12891559

[84] Lin JG Zheng SZ Chen AP. Curcumin attenuates the effects of insulin on stimulating hepatic stellate cell activation by interrupting insulin signaling and attenuating oxidative stress. Lab Invest. (2009) 89:1397–409. 10.1038/labinvest.2009.115 19841616 PMC2787823

[85] Shu T Pang M Rong LM Liu C Wang J Zhou W. Protective effects and mechanisms of salvianolic acid B against H[[sb]]2[[/s]]O[[sb]]2[[/s]]-induced injury in induced pluripotent stem cell-derived neural stem cells. Neurochemi Res. (2015) 40:1133–43. 10.1007/s11064-015-1573-6 25855584

[86] Nocella C Cammisotto V Bartimoccia S Castellani V Loffredo L Pastori D. A novel role of MMP2 in regulating platelet NOX2 activation. Free Radical Bio Med. (2020) 152:355–62. 10.1016/j.freeradbiomed.2020.03.033 32268176

[87] Yoon SO Park SJ Yoon SY Yun CH Chung AS Sustained production of H[[sb]]2[[/s]]O[[sb]]2[[/s]] activates pro-matrix metalloproteinase-2 through receptor tyrosine kinases/phosphatidylinositol 3-kinase/NF-κB pathway. J Biol Chem. (2002) 277:30271–82. 10.1074/jbc.M202647200 12058032

[88] Zhang ZB Wang QQ Ma J Yi XF Zhu YP Xi XW Feng YJ. Reactive oxygen species regulate FSH-induced expression of vascular endothelial growth factor via Nrf2 and HIF1α signaling in human epithelial ovarian cancer. Oncol Rep. (2013) 29:1429–34. 10.3892/or.2013.2278 23404377

[89] Timolati F Ott D Pentassuglia L Giraud M Perriard J Suter TM. Neuregulin-1 beta attenuates doxorubicin-induced alterations of excitation-contraction coupling and reduces oxidative stress in adult rat cardiomyocytes. J Mol Cell Cardiol. (2006) 41:845–54. 10.1016/j.yjmcc.2006.08.002 17005195

