# Supplementary material for: Antioxidant Effects and Potential Molecular Mechanism of Action of Limonium aureum Extract Based on Systematic Network Pharmacology
Source: Front Vet Sci. 2022 Jan 5;8:775490. doi: 10.3389/fvets.2021.775490 (PMC8767100; doi:10.3389/fvets.2021.775490)
Supplement: Supplementary Table S1 — The qRT-PCR primers for the hub genes. [file Table_1.DOCX]

| Primer | Sequence (5′→3′) | Length (bp) |
| --- | --- | --- |
| ERBB2-F | GTCAGAGATGCGACCCTCAG | 20 |
| ERBB2-R | GTGCCTTGGCAGACTTTCTTT | 21 |
| PRKCA-F | TACCAGGTGCCAGGAGATAGACAAG | 25 |
| PRKCA -R | AGACAGACAGACAGACAGACAGAGG | 25 |
| MMP2-F | AAGAACCGCTGCTCTAAGAAGTTGG | 25 |
| MMP2-R | GGAGGAATGTGAGGAATGGCTTGG | 24 |
| INSR-F | GGAGGAGGAGGCTTGACAGAGG | 22 |
| INSR -R | GGAAGAAGATGGCTGGAGAAGATGAC | 26 |
| PTGS2-F | TCATTGATGCCAAGACTGGACT | 22 |
| PTGS2-R | TTTCTTTGAACAGGTAGAGGCG | 22 |
| β-Actin-F | TGAGAGGGAAATCGTGCGTGAC | 22 |
| β-Actin-R | GCTCGTTGCCAATAGTGATGACC | 23 |

**Supplementary Table S1.** qRT-PCR Primers
