# Supplementary figures and images for: Antioxidant Effects and Potential Molecular Mechanism of Action of Limonium aureum Extract Based on Systematic Network Pharmacology
Source: Front Vet Sci. 2022 Jan 5;8:775490. doi: 10.3389/fvets.2021.775490 (PMC8767100; doi:10.3389/fvets.2021.775490)

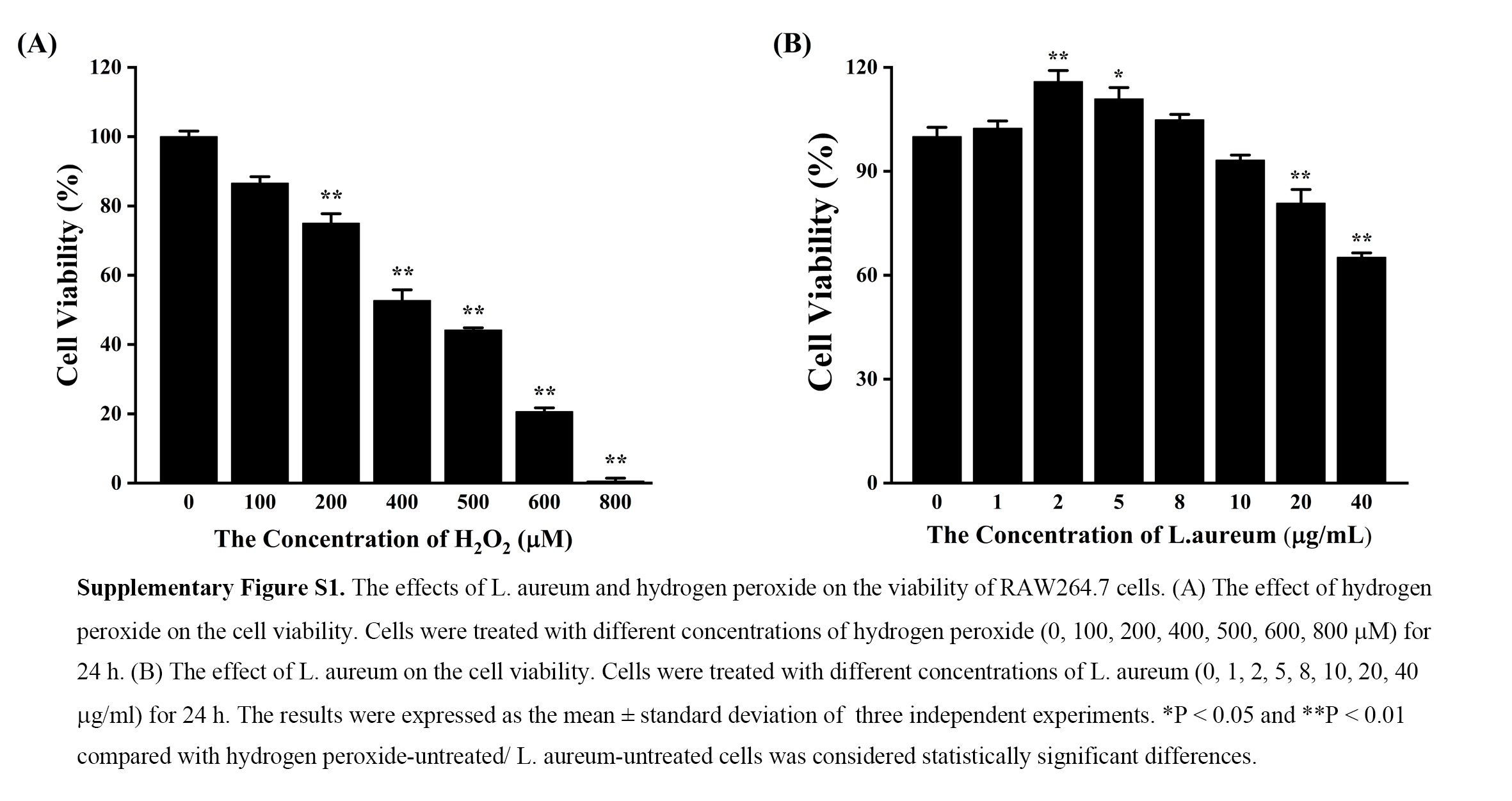

Supplement: Supplementary Figure S1 — The effects of L. aureum and hydrogen peroxide on the viability of RAW264.7 cells. (A) The effect of hydrogen peroxide on the cell viability. Cells were treated with different concentrations of hydrogen peroxide (0, 100, 200, 400, 500, 600, 800 qM) for 24 h. (B) The effect of L. aureum on the cell viability. Cells were treated with different concentrations of L. aureum (0, 1, 2, 5, 8, 10, 20, 40 qg/ml) for 24 h. The results were expressed as the mean + standard deviation of three independent experiments. *P < 0.05 and **P < 0.01 compared with hydrogen peroxide-untreated/L. aureum-untreated cells was considered statistically significant differences. [file Image_1.TIF]
